# Harnessing epithelial-mesenchymal plasticity to boost cancer immunotherapy

**DOI:** 10.1038/s41423-023-00980-8

**Published:** 2023-02-24

**Authors:** Yuanzhuo Gu, Zhengkui Zhang, Peter ten Dijke

**Affiliations:** 1 Oncode Institute and Department of Cell and Chemical Biology, Leiden University Medical Center, Einthovenweg 20, 2333 ZC Leiden, The Netherlands; 2 Institutes of Biology and Medical Science, Soochow University, Suzhou, 215123 China; 3 Oncode Institute and Division of Molecular Oncology and Immunology, The Netherlands Cancer Institute, Plesmanlaan 121, 1066 CX Amsterdam, The Netherlands

**Keywords:** Epithelial-to-mesenchymal plasticity (EMP), immune checkpoint blockade (ICB), immunotherapy resistance, tumor microenvironment (TME), Cancer microenvironment, Cell biology, Immunotherapy

## Abstract

Immune checkpoint blockade (ICB) therapy is a powerful option for cancer treatment. Despite demonstrable progress, most patients fail to respond or achieve durable responses due to primary or acquired ICB resistance. Recently, tumor epithelial-to-mesenchymal plasticity (EMP) was identified as a critical determinant in regulating immune escape and immunotherapy resistance in cancer. In this review, we summarize the emerging role of tumor EMP in ICB resistance and the tumor-intrinsic or extrinsic mechanisms by which tumors exploit EMP to achieve immunosuppression and immune escape. We discuss strategies to modulate tumor EMP to alleviate immune resistance and to enhance the efficiency of ICB therapy. Our discussion provides new prospects to enhance the ICB response for therapeutic gain in cancer patients.

## Introduction

The advent of immune checkpoint blockade (ICB) therapies, which (re)activate the T-cell mediated anti-tumor response [1], have demonstrated high clinical efficacy in some patients with difficult-to-treat cancers and even in patients with metastasis [2–7]. The US Food and Drug Administration (FDA) has approved eight immune checkpoint inhibitors thus far, including one cytotoxic T-lymphocyte antigen-4 (CTLA-4) mAb (ipilimumab), three programmed death-1 (PD-1) monoclonal antibodies (mAbs; nivolumab, pembrolizumab, and cemiplimab), three programmed death-1 ligand 1(PD-L1) mAbs (atezolizumab, durvalumab, and avelumab) and one lymphocyte activation gene-3 (LAG-3) mAb (relatlimab), and more ICBs are being tested in clinical trials [8–10]. Collective analysis of clinical studies for anti-PD-1 or anti-PD-L1 monotherapy revealed that the objective response rates (ORRs) vary across different cancer types and subtypes. In some tumors (e.g., melanoma, Merkel cell carcinoma, and Hodgkin’s lymphoma), ICB therapies achieve high ORRs ranging from ~40% to 70%, while in the majority of tumor types, the ORRs are less than 25% [8, 11–13]. Apparently, clinical success occurs in only a minority of patients; most patients do not respond to ICBs. Patients who never respond to treatment are described as having primary resistance, and patients who initially respond but fail to achieve a long-term, durable response and eventually relapse are described as having acquired resistance [14, 15]. Considering the unmet medical need, delineating the nature of the resistance mechanisms and optimizing the targeting of novel mediators could help refine and improve immunotherapy treatment goals [16–18].

Tumor epithelial–mesenchymal plasticity (EMP), which refers to the ability of cancer cells to undergo a dynamic and reversible morphological switch from epithelial-like cells to fully or partially mesenchymal-like cells (termed epithelial to mesenchymal transition (EMT), or from mesenchymal-like cells to fully or partially epithelial-like cells (termed mesenchymal to epithelial transition (MET) [19–22], has been identified as a critical determinant of cancer progression [23, 24]. EMP/EMT has been linked to cancer cell stemness, metastasis, chemotherapy resistance and immunosuppression [24–26]. Additionally, regarding ICB therapy, EMP has emerged as a potential mediator of immunotherapy resistance [27–31]. Here, we review recent emerging studies about the interplay between EMP and immunotherapy resistance, and in particular focus on underlying mechanisms. Importantly, we summarize the advanced methods used to identify EMP response during ICB therapy. Thereafter, we discuss how EMP is linked to ICB resistance via tumor-intrinsic and tumor-extrinsic mechanisms. Finally, we discuss the ongoing development of strategies to modulate EMP, as well as in vivo studies and preclinical trials, by combining other therapies with ICBs with the goal of improving immunotherapy.

## EMP

The concept of “epithelial-to-mesenchymal transformation” was first proposed by Elizabeth Hay in the late 1970s, which is a central process required for normal embryogenesis [32]. The cytokine transforming growth factor-β (TGF-β) was discovered as a potent inducer of EMT in 1989 [33]. After that, more and more molecular regulators (“drivers”) of EMT have been identified. In 1991, discoveries in the development field of *Drosophila melanogaster* found master regulators of EMT, such as transcription factors Snail (also known as SNAI1) and TWISTs (the basic helix–loop–helix factors) [34]. In 1994, Slug (Snail-related TF, also known as SNAI2) was found to induce EMT during chicken embryogenesis [35]. Savagner et al. showed that overexpression of Slug can convert epithelial carcinoma cells into mesenchymal status, which extended the concept of EMT to the study of cancer progression [36]. Subsequently numerous studies on EMT revealed linkage of EMT to different aspects of tumorigenesis, such as metastasis in 2000 [37], tissue fibrosis in 2002 [38] and cancer stem cells in 2008 [39]. Whereas EMT as a biological process in developmental biology was widely accepted, this was initially not the case in the field of cancer biology, where it was met with disbelief. Due to its transient characteristics and that mesenchymal cancer cells are difficult to separate from stromal fibroblasts, EMT is difficult to observe in clinical samples. At present, however, EMT in cancer is widely accepted, and it has been documented in biopsies from cancer patients, especially at the invasive front [40]. Research on EMT is expanding logarithmically over past 20 years and the concepts of EMT, MET and EMP are evolving. Recently, ‘the EMT International Association’ (TEMTIA) published a consensus statement [21], aiming to clarify the nomenclature and provide definitions and guidelines for this research field, which is helpful for the researchers.

**Fig. 1:**
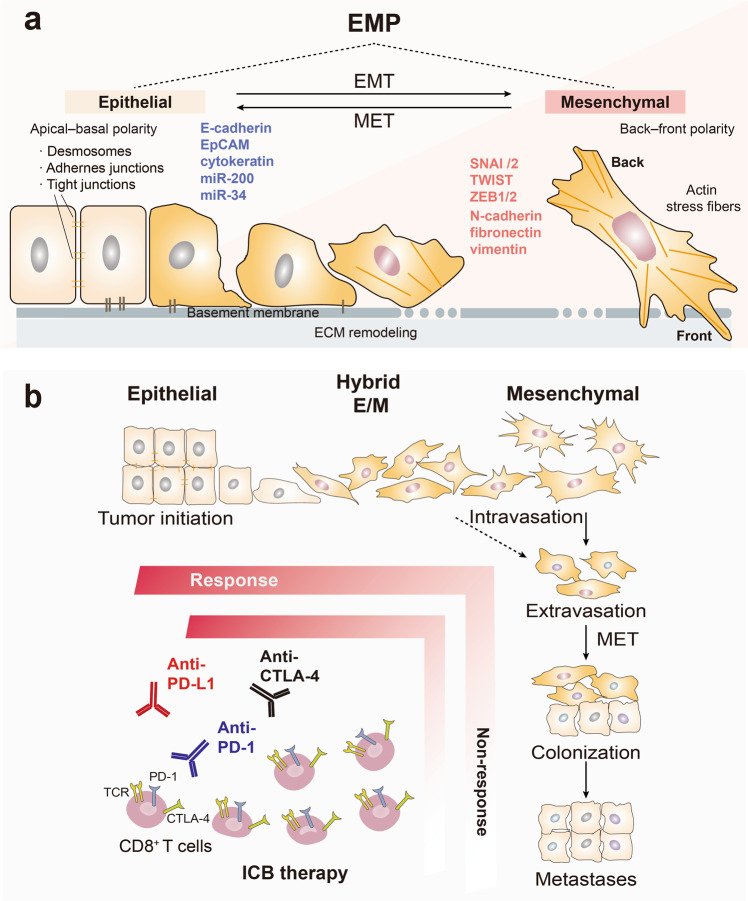
The relevance of EMP states and ICB response. **a** EMP refers to a broad spectrum of intermediate epithelial/mesenchymal (hybrid or partial E/M) phenotypic states between fully epithelial and fully mesenchymal of tumors with an active EMT or MET dynamic program. EMT is induced by a set of EMT-inducing transcription factors (EMT-TFs), like SNAI1/2, TWIST and ZEB1/2, that trigger epithelial cancer cells to undergo a series of molecular and morphological changes including tight junction dissolution, cell polarity alterations, cytoskeletal rearrangements, the loss of epithelial cell markers (e.g., E-cadherin, integrins, EpCAM, Claudin), take on appearance of mesenchymal cell phenotype, acquire increased motility, migration and apoptosis resistance, demonstrate elevated production of ECM components and emergence of mesenchymal cell markers (e.g., N-cadherin, vimentin, fibronectin). In contrast, mesenchymal cells can reverse to epithelial cells through activating a MET program. microRNAs such as miR-200 and miR-34 families are crucial for this process, which are regulated in double-negative feedback loops with the EMT-TFs ZEB1/2 and SNAI1/2, respectively. **b** Cancer cells exhibit different EMP states during cancer progression from primary tumors to distant metastases. Benign cells are initially transformed into cancerous epithelial cells. Cancer cells undergo dynamic phenotypic changes that are characterized by an epithelial state, an intermediate hybrid E/M hybrid state and a mesenchymal state through EMT or MET. Cancer cell EMP is a critical determinant in regulating immune escape and resistance to ICB therapies, e.g., anti-PD-1, anti- PD-L1 and anti-CTL-4. CTL-4 cytotoxic T-lymphocyte-associated protein 4, E/M epithelial/mesenchymal, EMT epithelial to mesenchymal transition, EMP epithelial mesenchymal plasticity, ICB immune checkpoint blockade, MET mesenchymal to epithelial transition, MiR micro RNA, PD-1 programmed cell death protein 1, PD-L1 programmed death-ligand 1

During the development of cancer, cancerous epithelial cells undergo multiple processes to transform from benign into malignant cells, including local outgrowth and acquisition of a more mesenchymal phenotype that confers the ability to migrate and invade into the neighboring tumor microenvironment (TME), disseminate into the circulation, colonize distant tissues, and undergo local outgrowth at these sites, accompanied by MET [41]. During these processes, cancer cells exhibit dynamic plasticity and are present in an epithelial state, intermediate hybrid epithelial/mesenchymal state (hybrid E/M or partial EMT) or mesenchymal state [21, 42–47]. Upon EMT activation, cancer cells typically shed the expression of epithelial markers, such as E-cadherin, epithelial cell adhesion molecule (EpCAM), lose their apical–basal polarity and cell-cell adhesion properties, demonstrate increased levels of mesenchymal markers such as filamentous actin stress fibers, N-cadherin, vimentin and exhibit an enhanced capacity to migrate and invade. The EMT program is regulated by a core set of EMT-inducing transcription factors (EMT-TFs), like SNAI1/2, TWIST1/2, and the zinc finger E-Box binding homeobox factors (ZEB1 and ZEB2). In contrast, mesenchymal cells can reverse to epithelial cells through activating MET program. microRNAs such as miR-200 and miR-34 play important role in this process, which suppress EMT-TFs ZEB1/2 and Snail respectively to form a negative feedback (Fig. 1a) [21]. Now it is generally accepted that, although EMT is activated in many cancer types, it is rarely fully executed in tumor cells, and end-stage markers such as vimentin are often not expressed. Numerous studies support the notion that tumor cells can express both epithelial and mesenchymal markers, exhibiting hybrid epithelial/mesenchymal (E/M) phenotypes (hybrid EMT) [48–50]. A broad spectrum of intermediate E/M phenotypic states between fully epithelial and fully mesenchymal has been recently demonstrated [43, 44, 46, 51]. The term ‘epithelial-mesenchymal plasticity’ (EMP) better describes the ability of cells to adopt hybrid E/M features and to interconvert between EMT and MET [21, 50, 52].

Such kinds of interconvertible, dynamic phenotypic plasticity and heterogeneity enables tumor cells possessing modifiable traits to adapt to diverse microenvironments [53–55]. The hybrid E/M state was reported to be sufficient for the tumorigenicity of basal breast cancer cells, while fully mesenchymal or fully epithelial populations exhibited poor tumor-initiating ability [42, 44]. Moreover, hybrid E/M states, rather than the fully epithelial and mesenchymal states, were found to be associated with poor clinical prognosis in diverse cancers, possibly due to the hybrid E/M features of circulating tumor cells (CTCs) [56–61]. Li et al. used a dual recombination system to perform lineage tracing of estrogen receptor (ER)-negative mammary luminal cells undergoing EMT in mouse mammary tumor virus polyoma middle tumor-antigen (MMTV-PyMT) transgenic mice and revealed that EMT was not activated in the tumor cells at the early stages, but was activated in the later stages of tumorigenesis, and that metastasis-initiating cells underwent EMT during metastasis [62]. In another study using a genetic lineage tracing system, similar results were obtained; partial EMT but not full EMT in cells was associated with lung metastasis, while both partial and full EMT in cells contributed to chemotherapy resistance [63]. The above findings demonstrate that partial and full EMT is probably the most relevant form in tumors, which can place cancer cells in a dynamic window that may endow them with plasticity, and thereby promote cell invasion and insensitivity to chemotherapy but also to immunotherapy (Fig. 1b). In the forthcoming sections, we provide a critical overview of studies reporting (causal) links between EMP states as determinants for the efficiency of the ICB therapy response.

## Advanced methods to study ICB response of EMP: Are mesenchymal-like tumor cells more therapy-resistant?

**Fig. 2:**
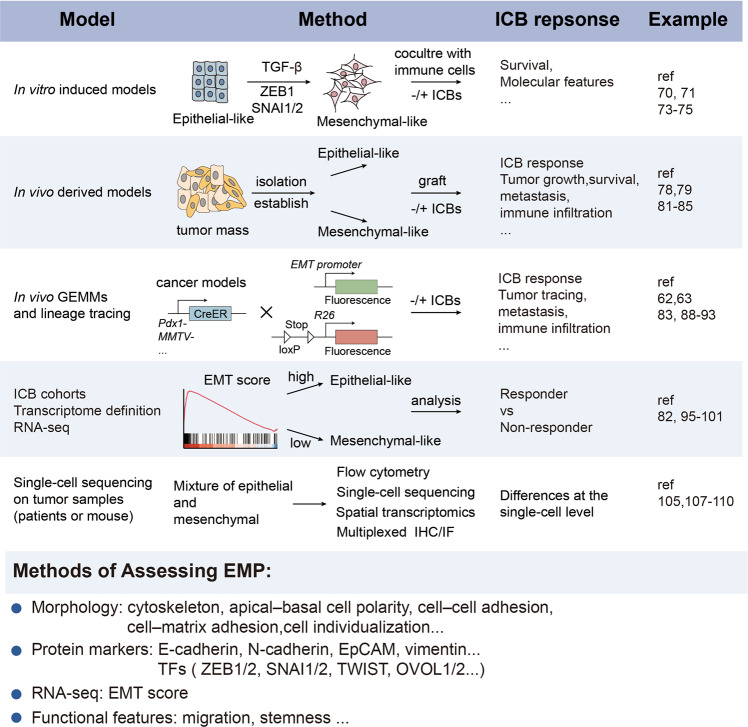
Advanced methods to study the link between EMP and the ICB response. The schematic diagram illustrates the advanced methods and models used to clarify the relationship between the ICB response and diverse EMP states in tumors. (1) In vitro induced models: 2 types of cancer cells (epithelial-like or mesenchymal-like) driven by EMT-inducing growth factors such as TGF-β or EMT-TFs are established, and then cocultured with immune cells to assess tumor growth and molecular features; (2) in vivo derived models: epithelial-like or mesenchymal-like cancer cells are isolated from bulk tumors based on cell surface markers or in vitro cell lines are established for transplantation and assessment of the response to ICB therapies; (3) analysis of available ICB cohorts: differences in the ICB response between EMP groups are determined based on the EMT score; (4) in vivo GEMMs and lineage tracing: a lineage tracing system is used to study dynamic EMP and ICB response in GEMMs; (5) single-cell sequencing on tumor samples from patients or mice: tumors treated with ICBs are analyzed at the single-cell level. Several methods are usually used to assess EMP, including analyses of cell morphology, molecular markers, signatures and functional changes. CreER tamoxifen-dependent Cre recombinase, E-cadherin epithelial cadherin, EMP epithelial mesenchymal plasticity, EMT epithelial to mesenchymal transition, GEMM genetically engineered mouse model, ICB immune checkpoint blockade, IHC immunohistochemistry, IF immune fluorescence, MMTV mouse mammary tumor virus, OVOL ovo-like 1 transcription factor, TGF-β transforming growth factor-β, TFs transcription factors

Studies in in vitro cultured cell lines, in vivo cancer models and clinical ICB datasets, have shown that the development of resistance to immunotherapy is associated with EMP [27–31] (Fig. 2).

### In vitro models

Certain epithelial cancer cell lines can be manipulated to acquire different EMP states upon stimulation with external factors, such as TGF-β, and hepatocyte growth factor (HGF) or by ectopic expression of EMT-TFs [44]. The responses of cancer cells or tumoroids with diverse EMP states (characterized by the expression of functional epithelial and mesenchymal markers) to immune cells were evaluated using immune-tumor cell coculture systems [64–68] or even an ex-vivo tumor fragment platform [69]. Kudo-Saito and colleagues established murine and human melanoma cells with EMT-like features using transduction with an expression plasmid encoding Snail. They demonstrated that Snail^+^ melanoma cells cocultured with spleen cells induced regulatory T cells and impaired dendritic cells (DCs) in vitro, and mediated resistance to gp70 peptide-pulsed DCs transfer therapy in vivo [70]. Similarly, compared with the parental cells, MCF7 human breast cancer cells that acquired mesenchymal features through overexpression of wild-type Snail, expression of a constitutively activated Snail mutant (Snail-S6A) or long-term tumor necrosis factor α (TNFα) treatment, showed more resistance toward cytotoxic T lymphocyte (CTL)-mediated killing [71]. Reduced expression of major histocompatibility complex (MHC) molecules (MHC)-I was observed in PC3 and DU145 prostate cancer cells that were engineered to ectopically express Snail or were treated with TGF-β and epidermal growth factor (EGF) [72]. Of note, aberrant expression of brachyury, a T-box transcription factor, in human cancer cells drove EMT and reduced cancer cell susceptibility to antigen-specific T-cell and natural killer (NK) cell-mediated killing [73–75]. On the contrary, EMT-TFs or related inducers initiate production of thrombospondin-1 (TSP1), TGF-β, interleukin-2 (IL-2), interleukin-6 (IL-6) and chemokine (C-X-C motif) ligand 20 (CXCL20) to recruit immunosuppressive cells and upregulate levels of immune checkpoint molecules on tumor cells with mesenchymal state, leading to an immunosuppressive tumor microenvironment [31, 76]. These results suggest that mesenchymal features can make tumor cells more resistant to immunotherapy. However, it is important to note that there are many different EMP states; EMT is not simply biphasic. In the studies above, it is unclear which particular EMP states contributed to the immune resistant phenotypes. In contrast to the concept that cells with mesenchymal-like states are more resistant to CTL-mediated killing, Lopez-Soto et al. found that colorectal cancer cells with Snail-induced EMT were more susceptible to NK cell-mediated killing because of increased expression of NK group 2, member D ligands (NKG2DLs) in these cells [77].

### In vivo derived xenograft models

Tumor cell lines with different EMP states can be derived from in vivo generated tumors [78–80]. Gibbons and colleagues generated a panel of epithelial and mesenchymal cancer cells (from a murine *K-ras*^*LA1/+*^*p53*^*R172HΔg/+*^ *(KP)* lung cancer model) with distinct EMP features [81]. These tumor-derived cell lines were transplanted into immunocompetent syngeneic mice, and mesenchymal transition was found to impair the function of CD8^+^ tumor-infiltrating lymphocytes (TILs) [82]. Using two epithelial cell lines (pB-2, EpCAM^high^ Snail^low^) and two mesenchymal cell lines (pB-3, EpCAM^low^ Snail^high^) derived from the MMTV-PyMT breast cancer model, the Weinberg group demonstrated that the mesenchymal cell lines have an immunosuppressive phenotype and that mesenchymal tumors were more refractory to anti-CTLA-4 treatment [83, 84]. Importantly, they discovered that even a very small proportion of mesenchymal cells can protect epithelial cells against immune attack and confer resistance to anti-CTLA-4 therapy [84]. In a more recent paper, they further demonstrated that quasi-mesenchymal tumor cell-derived factors, i.e., ecto-5’-nucleotidase (CD73), colony stimulating factor 1 (CSF1), and osteopontin (SPP1, a secreted phosphoprotein) cross-protect epithelial tumor cells against immune attack in mixed tumor and that blocking these factors partially or completely sensitizes refractory tumor cells to anti-CTLA-4 therapy [85]. It will be interesting to investigate whether there are (in addition to the secretome of mesenchymal tumor cells) other factors, such as pharmacologically targetable membrane proteins, that contribute to increased immune resistance. It’s important to note that in order to investigate the direct effect of EMT in immunotherapy response using those genetic models, other confounding factors, such as cell cycle, apoptosis/death and stemness, to EMT [86, 87] that can act in concert with EMT need to be considered.

### Genetically engineered mouse models (GEMMs) and lineage tracing

To monitor the EMP state in vivo, several powerful GEMMs and lineage tracing models have been developed and used to trace individual cancer cells during metastasis [20]. Epithelial-specific markers (such as E-cadherin), mesenchymal-specific markers (such as fibroblast specific protein-1 (Fsp1), vimentin and N-cadherin) and EMT-TFs (Snail and Slug) have frequently been used to trace the EMP state in mice cancer models in vivo. For example, one pioneering model is *Fsp1-Cre*: *Rosa26*^loxP^-*stop*-*lacZ* transgenic mice, in which Cre enzyme expression in mammary stromal cells is driven by the *Fsp-1* promoter and the conditional *Rosa26*^loxP^ reporter locus contains a “floxed stop cassette” located upstream of the *lacZ* gene. This model was used to elucidate the timing and functional significance of EMT in MMTV-PyMT initiated breast cancer in vivo [88]. In a further-improved model [89], the reporter gene lacZ was replaced by green fluorescent protein (GFP) or by switchable red fluorescent protein (RFP) and GFP, which allows RFP^+^ epithelial tumor cells to be converted to GFP^+^ cells following *Fsp1-Cre* activation [90]. To study whether the origin of cancer cells affects EMT in skin tumors, Latil et al. generated a two-lineage-specific CreER model in which tumor cells initiated by *KRas*^*G12D*^ expression and *p53* deletion can be individually labeled with yellow fluorescent protein (YFP) under *K14*^*CreER*^ and *Lgr5*^*CreER*^ expression in the interfollicular epidermis and hair follicle stem cells [91]. In addition to the models with indirect Cre-mediated labeling, models have been established with direct knock-in of fluorescent reporter proteins (YFP or CFP) into EMT marker genes (such as Snail, Slug and N-cadherin) downstream of the internal ribosome entry site (IRES) to endogenously label tumor cells [83, 92].

Remarkably, dual reporter genetic mouse models, which can allow monitoring of the dynamic and transient EMP states, have also recently been developed. Li et al. generated an elegant, novel model combining two recombinase-mediated systems (*Cre/loxP* and *Dre/rox*) using either *N-cadherin-Dre* or *vimentin-Dre* to monitor cells undergoing transient EMT in the lung metastases of mammary tumors [62]. In addition, the Christofori group employed another tamoxifen-inducible dual recombinase system (*Flpo/Frt* and *Cre/loxP*) using either *Tnc-CreER*, an early EMT marker, or *N-cadherin-CreER*, to study partial and full EMT during breast cancer metastasis [63]. In a spontaneous pancreatic ductal adenocarcinoma (PDAC) model, mice with mesenchymal cell reporter expression driven by *aSMA-Cre*, a marker for partial EMT, or *Fsp1-Cre* were generated to monitor the partial EMT program [93]. These GEMMs and lineage labeling systems have enabled detailed studies of the dynamics of EMP during tumor development. However, to date, there have been no reports using these models to investigate the ICB response of tumors with different EMP states, and such reports are eagerly awaited.

### Clinical ICB cohorts

Analysis of human cancer datasets also demonstrated that the EMT score (based on different EMT signatures) [94] strongly correlated with the immunosuppression signatures across different types of solid cancers [95–98]. The EMT score was found to be inversely associated with CD8^+^ T-cell infiltration in lung cancer [82, 96]. In addition, tumors with high mesenchymal EMT scores exhibited high levels of PD-L1 [82, 95] and other immune checkpoints, including PD-1, CTLA-4, tumor necrosis factor receptor OX40 ligand (OX40L), and programmed death-1 ligand 2 (PD-L2) [95]. Furthermore, studies of ICB-treated patients support the concept that EMP (e.g., the EMT gene signature, or a mesenchymal phenotype) is a critical mediator of ICB resistance [98–100]. The EMT signature is significantly enriched in nonresponding tumors across multiple cancer subtypes before treatment [98], and patients with these tumors likely express higher levels of genes linked with the mesenchymal phenotype (such as *ZEB1, AXL receptor tyrosine kinase, ROR2 (receptor tyrosine kinase like orphan receptor 2), WNT5A (wnt family member 5* *A)*, and *TWIST2*) [98, 99]. In some urothelial cancer patients with T-cell-infiltrated tumors, high EMP/stromal features were found to impair the response to a PD-1 inhibitor (nivolumab) and shortened progression-free and overall survival (OS) times [101]. Intriguingly, the EMP status did not universally correlate with OS in most cancer subtypes [102]. Ovarian cancer, gastric cancer, pancreatic cancer and glioblastoma patients with a low EMT score showed better OS, while no correlation between the EMT score and OS was observed in acute myeloid leukemia, colorectal cancer and lung cancer. These results indicate that the EMT score is unlikely to be the sole prognostic factor determining OS [102]. Notably, the bulk sequencing data contained data for other nontumor cells that may have affected the EMT scores. For example, contamination by stromal cells increases the mesenchymal score [103, 104]. Therefore, when using bulk sequencing data, users should not only focus on the EMT score but also consider tumor cell population purity and double check the correlation analysis results.

### Single-cell sequencing of tumor samples

Single-cell RNA sequencing (scRNA-seq) offers remarkable opportunities to systematically investigate the heterogeneity of tumors and TME in different cancer types, as has been demonstrated in studies on glioblastoma [105], breast cancers [106, 107] and liver cirrhosis [108]. Deshmukh et al. performed scRNA-seq on normal human breast MCF10A cells stimulated with TGF-β1 and observed diverse EMP states with sequential and parallel activation of EMT signaling pathways. By analyzing the gene expression profiles from the scRNA-seq data, this group demonstrated that gene signatures more aligned with the mesenchymal state are associated with poorer survival rates in cancer patients [109]. In another study, scRNA-seq profiling was performed in a 2D in vitro cultured MCF10A and human mammary epithelial cells (HuMECs) system in which the inner and outer cells undergo a spontaneous spatially determined EMT program in the presence or absence of TGF-β. The results identified different classes of cells across the full spectrum of EMP states [110]. In addition to the studies in cell lines, Sehgal et al. conducted dynamic scRNA-seq on anti-PD-1 treated murine organotypic tumor spheroids, and identified an immunotherapy resistant subpopulation was positive for Snail and stem cell antigen 1 (Sca-1) expression and exhibited hybrid epithelial mesenchymal features [111]. scRNA-seq analysis of glioblastoma multiforme (GBM) revealed that the mesenchymal-like subtype of GBM evades immune clearance by activating a myeloid-associated transcriptional program, which leads to increased recruitment of tumor-associated macrophages(TAMs) [112]. Carstens et al. performed scRNA-seq on murine pancreatic ductal adenocarcinoma tumors in an in vivo genetic cancer model and found a spectrum of epithelial and mesenchymal cancer cells. It was noteworthy that there were fewer CD8^+^ T cells in the mesenchymal region, which may indicate a poor response to ICB therapy [113]. In future studies, single-cell analysis of tumors treated with ICBs will help us to better understand how the EMP states affects the response to immunotherapy [114–116].

## EMP and ICB tumor-intrinsic resistance mechanisms

**Fig. 3:**
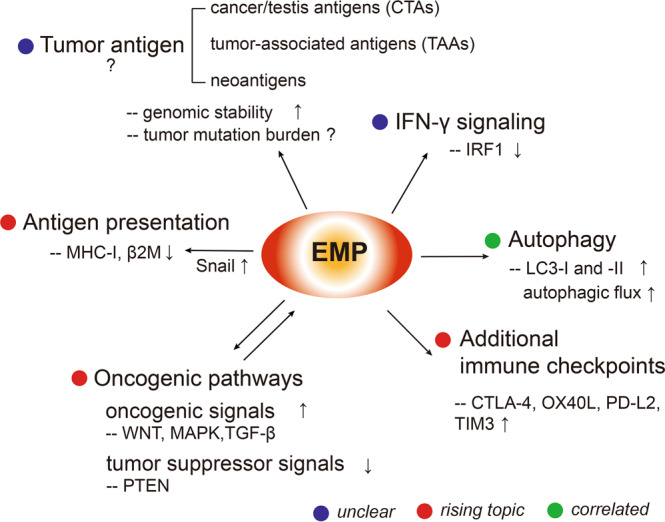
EMP and ICB tumor-intrinsic resistance mechanisms. EMP may contribute to ICB resistance by modulating multiple tumor-intrinsic mechanisms, such as inducing increased autophagy, additional immune checkpoints, aberrant oncogenic/tumor suppressor signals, decreased sensitivity to IFN-γ and defects in antigen presentation. Their current research stages were annotated with the assigned colors. Blue stands for “unclear”, meaning further mechanistic work is needed; red stands for “rising topic”, meaning the mechanism is revealed or emerging; green stands for “correlated”, meaning the topic is related to EMP and immune resistance, but should be confirmed by more functional studies. β2M β2 microglobulin, CTLA-4 cytotoxic T-lymphocyte antigen-4, EMP epithelial mesenchymal plasticity, IRF1 interferon regulatory factor 1, LC3 microtubule-associated protein 1 A/1B-light chain 3, MAPK mitogen-activated protein kinase, MHC-I major histocompatibility complex I, OX40L tumor necrosis factor receptor OX40 ligand, PTEN tumor suppressor phosphatase and tensin homolog, PD-L2 programmed death-2 ligand, Snail snail family transcriptional repressor 1, TIM3 T-cell immunoglobulin and mucin domain-containing-3, TGF-β transforming growth factor β, WNT wnt family member protein

**Fig. 4:**
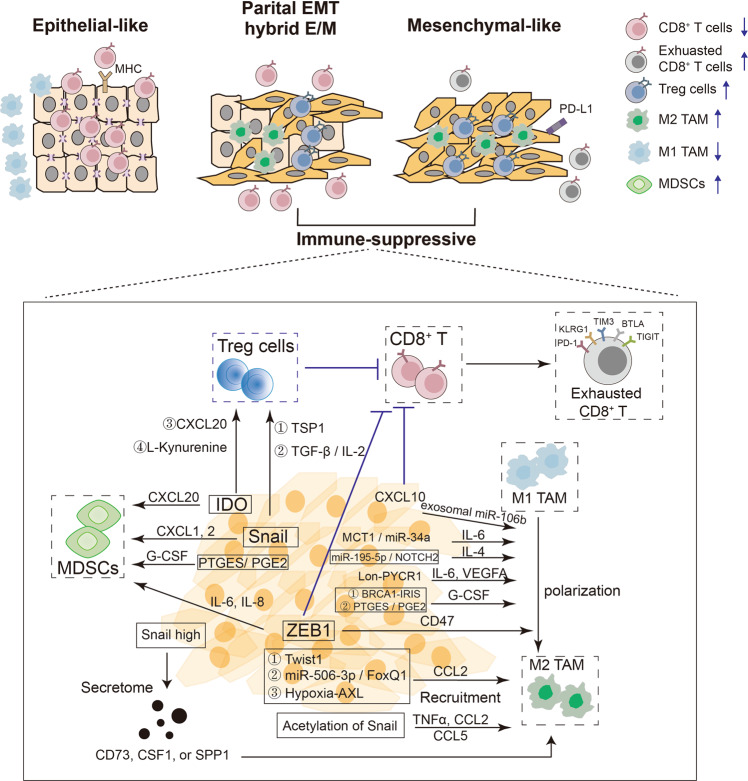
EMP impacts cancer cell-immune cell interactions, shaping the immunosuppressive TME. Cancer cell clusters that exhibit more epithelial-like, more mesenchymal-like or mixed epithelial-like and mesenchymal-like phenotypes interact differently with different types of immune cells in the TME. Compared with epithelial-like tumors, tumors with a partial EMP or mesenchymal-like phenotypes generate an immunosuppressive TME, in which EMP-TFs or their upstream activators mediate the secretion of specific cytokines or chemokines to recruit immunosuppressive cells (Tregs, TAMs, and MDSCs) and thereby exclude CD8^+^ CTLs to the periphery or dampen CD8^+^ T cells with exhausted CD8^+^ T cell features, such as increased expression of PD-1, KLRG1, TIM3, BTLA or TIGHT. AXL receptor tyrosione kinase of the TAM family, BRCA1-IRIS in-frame reading of intron 11 splice variant, BTLA B and T lymphocyte attenuator, CD8 cluster of differentiation 8, E/M epithelial/mesenchymal, EMT epithelial to mesenchymal transition, EMP epithelial mesenchymal plasticity, CCL chemokine (C-C motif) ligand, CXCL chemokine (C-X-C motif) ligand, IDO indoleamine 2,3-dioxygenase, IL interleukin, G-CSF granulocyte-colony stimulating factor, KLRG1 killer cell lectin like receptor G1, MDSCs myeloid-derived suppressor cells, PD-1 programmed cell death protein 1, PTGES/PGE2 prostaglandin E synthase/prostaglandin E2, Snail Snail family transcriptional repressor 1, SPP1 ostepontin, TAMs tumor associated macrophages, TIGIT T-cell immunoglobulin and ITIM domain, TIM3 T-cell immunoglobulin and mucin domain-containing-3, TGF-β transforming growth factor β, TF transcription factor, TNF tumor necrosis factor, TME tumor microenvironment, Treg regulatory T cell, VEGF vascular endothelial growth factor, ZEB1 zinc finger E-Box binding homeobox factor 1

The mechanisms responsible for the failure to respond or achieve a durable response to ICB therapy in most patients have been an increasingly much researched topic, which have been well highlighted by other excellent reviews [7, 14, 15, 17, 18, 117, 118]. These mechanisms can be generally classified into two categories: (1) tumor-intrinsic mechanisms, such as loss of tumor neoantigen expression, defects in the antigen presentation machinery, deficient interferon-γ (IFN-γ) signaling, aberrancies in oncogenic/tumor suppressor pathways that mediate immune escape and created additional inhibitory checkpoints; and (2) tumor-extrinsic mechanisms, which confer an immunosuppressive microenvironment, including reductions in the function or number of CD8^+^ effector T cells (Teff cells), induction of myeloid-derived suppressor cells (MDSCs), an increase in the number of regulatory T cells (Tregs) or M2-like TAMs, production of immunosuppressive cytokines (e.g., interleukin-8 (IL-8), TGF-β or pro-tumorigenic factors (e.g., vascular endothelial growth factor (VEGF)), and the activity of other cells in the TME, e.g., CD4^+^ T cells, NK cells, stromal cells, and DCs. Notably, in addition to the proposed mechanisms, many other cases mechanisms have been identified, and other mechanisms remain unknown [15, 119–127]. Below, we discuss their relevance to EMP and immune evasion, from a tumor-intrinsic and -extrinsic perspective (Figs. 3, 4).

### Tumor antigens

There’re three broad classifications of tumor antigens which can be recognized as immune targets by T cells: (1) cancer/testis antigens (CTAs); (2) tumor-associated antigens (TAAs); (3) neoantigens [128]. Previous studies have revealed that aberrant expression of oncogenic CTAs, such as MAGE-D4B (MAGE family member D4B), CT45A1 (cancer/testis antigen family 45 member A1), PRAM1 (PML-RARA regulated adaptor molecule 1), can contribute to stimulate EMT via altering the expression of EMT related genes [129]. Epigenetic regulation including DNA methylation and histones modification has been shown as the most commonly employed mechanisms by which the transcriptional regulation of CTA genes is controlled [130, 131]; the role of EMP in influencing CTA remains to be investigated. TAAs can be subclassed as (1) differentiation antigens, which are normal proteins overexpressed leading to tumorigenic phenotypes, such as B-cell lineage-specific CD19 [132] and (2) overexpressed antigens, which are consistently overexpressed by tumors, such as p53 [133], ERBB2 [134]. A multiple-omics profiling study of chronic lymphocytic leukemia revealed that a mesenchymal phenotype was associated with high genomic stability and resistance to DNA damage, which reduced the therapeutic benefit of rituximab, a chimeric mAb targeting the pan-B-cell marker CD20 [135]. In another study, however, the tumor mutation burden did not correlate with the EMT signature [98]. Neoantigens are aberrant polypeptides, which can arise from genomic mutations in tumor cells. The sources of tumor neoantigens are summarized by a recent review [136], and include genomic variants, genetic fusion, selective splicing of transcriptome, RNA editing and mutations in non-coding regions. Neoantigen-specific CD8^+^ T cells expand in response to ICB therapy [137, 138], and high neoantigen or mutation burden is generally predictive of response to checkpoint blockade. However, the research related to the interplay between EMP and neoantigens is still limited.

### Antigen presentation machinery

In the MMTV-PyMT murine breast cancer model, mesenchymal-like tumors (Snail^high^) had lower major histocompatibility complex (MHC)-I protein levels, decreased β2 microglobulin (β2M) expression, and increased PD-L1 expression compared to epithelial-like tumors (Snail^low^) [84]. On the contrary, β2M induces EMT [139], may participate in a negative feedback. These features impaired the recognition and targeting of cancer cells via activated Teff cells, leading to escape from immune system surveillance. Consistently, in prostate cancer cells, TGF-β- and epidermal growth factor (EGF)-induced EMT phenotypes were found to be associated with downregulation of human leukocyte antigen (HLA)-I [72]. Nintedanib, a triple angiokinase inhibitor, inhibited the EMT process in a metastatic 4T1 tumor model, and was found to upregulate MHC-I expression to enhance efficacy of PD-L1 blockade [140].

### PD-L1 and additional inhibitory checkpoints

Lou et al. observed that an EMT signature was associated with increased expression of various immune inhibitory ligands and receptors (e.g., PD-L1, PD-1, CTLA-4, T-cell immunoglobulin mucin-3 (TIM-3), LAG-3 and B7 Homolog 3 (B7-H3)) in lung adenocarcinomas [96]. Pulmonary adenocarcinoma tissues with mesenchymal and epithelial mesenchymal phenotypes had significantly higher PD-L1 staining than those with epithelial and unspecified phenotypes [141]. Increased PD-1, PD-L1, PD-L2 and CTLA-4 expression was also found in melanomas with robust EMT induced by a lack of epithelial splicing regulatory protein 1 (ESRP1) expression [142]. Immunohistochemistry (IHC) analysis revealed a significant correlation between PD-L1 expression and EMT status in thymic carcinoma [143]. In addition, activation of ZEB1 or repression of miR-200-induced EMT directly upregulated PD-L1 expression in human and murine non-small-cell lung cancer (NSLC) cell lines even in the absence of IFN-γ [82]. Moreover, PD-L1 expression was increased in macrophages and DCs cocultured with mesenchymal oral squamous cell carcinoma cells in vitro [144].

### IFN-γ signaling

Mesenchymal-like cells may lose sensitivity to IFN-γ signaling via suppression of interferon regulatory factor 1 (IRF1) expression [145]. Mechanistically, upregulated ZEB1, by interacting with C-Terminal Binding Protein (CtBP), suppresses the transcription of IRF1 through binding to its promoter. In addition, IFN-γ can promote EMP in human nasal epithelial cells via p38/ERK mitogen activated protein (MAP) kinase signaling [146] and in prostate cancer via microRNA (miRNA) processing [147]. Chemically, physically, and genetically engineered EMT hinder interferon-γ-dependent immunosurveillance in lung cancer cells via attenuating IFN-γ-induced IRF1 transactivation [148].

### Autophagy

Autophagy has been identified as a major tumor intrinsic resistance determinant for CTL-mediated killing [149–152]. Consistent with this notion, EMT was also found to escape CTL-mediated killing through autophagy induction [71, 153]. In the presence of the autophagy inhibitor chloroquine, microtubule-associated protein 1 light chain 3 (LC3)-II accumulated in mesenchymal breast cancer cells. These data suggest that autophagic flux is activated during EMT. Furthermore, inhibition of autophagy sensitized mesenchymal tumor cells to CTL-mediated killing [71, 153].

### Aberrant oncogenic/tumor suppressor signals

A series of studies across different types of cancer have indicated that augmented WNT/β-catenin signaling [154, 155], increases mitogen-activated protein kinase (MAPK) signaling [156] and that loss of tumor suppressor phosphatase and tensin homolog (PTEN) expression [149] renders tumors resistant to ICB, mainly because these changes cause alterations in the immune cell composition and cytokine-chemokine profiles [117]. In addition, the activation of oncogenic or inactivation of tumor-suppressive signaling pathways plays important roles in triggering and/or maintaining EMT [157]. Some of the signals also affect cell cycle, apoptosis and tumor stemness, which are consequences of EMT or further promote EMT. For example, tumors with RAS and p53 mutations, frequently observed in lung and pancreatic cancers, are quiescent in the process of metastasis, but they present an active EMT program [158]. And the mutations of p53 and KRAS in lung cancer cells could activate nuclear factor (NF)-κB pathway to promote tumorigenesis via suppression of apoptosis [86]. In the same mouse model, TGF-β and RAS-MAPK signals acted jointly through SMAD family members (SMADs) and Ras responsive element binding protein 1 (RREB1) transcription factors trigger EMT to promote tumor progression [159]. These alterations in oncogenic/tumor suppressor genes may be responsible for EMT-mediated immune evasion, but at the same time, apoptosis, DNA damage repair defects and other phenotypes are also triggered together with EMT. Therefore, how to evaluate the contribution of ICB therapy resistance triggered by either cell cycle, apoptosis, stemness or EMT is difficult and relies on more specific models.

## EMP and ICB tumor-extrinsic resistance mechanisms: an immunosuppressive TME

EMP is dynamically involved in the interaction with immune cells [54, 160]. Specifically, EMP may influence the immunosuppressive TME by regulating the numbers and functions of CD8^+^ T cells, exhausted CD8^+^ T cells, NK cells, Tregs, MDSCs, M1 TAMs, M2 TAMs and other cell types [76, 96, 161] (Fig. 4). EMT-induced TFs and their activated gene targets have extensive effects on immune cells in the TME, as shown in Fig. 4 and Table 1.

**Table 1:** The Roles of EMP-induced TFs or activated genes in the immunosuppressive TME

Immune cells	How	Cancer types	Reference
Tregs↑, CD8^+^ T cells ↓	Snail induced the generation of CD4^+^ Foxp3^+^ Tregs and impaired the generation of MHC-II^lo^ IDO-expressing regulatory DCs partially through TSP1 production.	Melanoma	[70]
	The a protein kinase c (PKC)-ι/P-Sp1/Snail axis resulted in generation of CD4^+^ CD25^-^ Tregs partially by mediating TGF-β1 and IL-2.	Cholangiocarcinoma	[161]
	CCL20 derived from hepatoma cells undergoing EMT induced IDO expression in monocyte-derived macrophages, which in turn suppressed T-cell proliferation and promoted the expansion of Tregs.	Hepatocellular carcinoma	[167]
	L-kynurenine induced IDO1 expression, decreased E-cadherin expression and increased N-cadherin expression. N-cadherin-positive tumor areas harbored fewer intraepithelial cytotoxic CD8^+^ T cells and more Tregs (CD4^+^/FOXP3^+^).	Prostate cancer	[166]
	ZEB1 decreased the infiltration of CD8+ T cells via CXCL10 secretion, independent of β-catenin activation.	Melanoma	[164]
	Arid5a stabilized IDO1 and CCL2 RNAs, which promoted the infiltration of granulocytic MDSCs and regulatory T cells.	Pancreatic ductal adenocarcinoma	[125]
M2 TAM recruitment	Acetylation of Snail induced the transcription of TNFα, CCL2 and CCL5, which promoted the recruitment of TAMs.	Head and neck cancer	[168]
	TWIST1 recruited stromal macrophages through CCL2 induction.		[169]
	The miR-506-3p/FoxQ1 axis induced TAM recruitment through CCL2 production.	Colorectal cancer	[170]
	The AXL tyrosine kinase was required for hypoxia-induced EMT and CCL2 induced macrophages behaviors.	HER2^+^ mouse model of breast cancer	[171]
	Snail^high^ cells secreted CD73, SPP1, or CSF1 to directly or indirectly recruit M2 TAMs.	Breast cancer	[85]
TAM polarization (M1-M2)	Exosomal miR-106b derived from EMT-colorectal cancer (CRC) cells induced M2 macrophage polarization.	Colorectal cancer	[172]
	The MCT-1/miR-34a axis induced M2 macrophage polarization through stimulating IL-6 secretion.	Triple-negative breast cancer (TNBC)	[285]
	The miR-195-5p/NOTCH2 axis inhibited M2-like TAM polarization by suppressing GATA3-mediated interleukin-4 (IL-4) secretion.	Colorectal cancer	[286]
	Lon-PYCR1 promoted EMT via ROS-dependent p38 MAPK and NF-κB signaling, stimulating M2 macrophage polarization through IL-6 and VEGF-A.	Oral squamous cell carcinoma	[173]
	BRCA1-IRIS- (an alternatively spliced BRCA1 product) overexpressing cells recruited M2 macrophages through secretion of GM-CSF.	TNBC	[287]
	The PTGES/PGE2 axis induced MDSC recruitment by G-CSF, and induced M2 macrophage polarization.	Non-small cell lung cancer (NSCLC)	[288]
	ZEB1 mediated induction of CD47 expression, induced M2 polarization of adjacent macrophages.	K-Ras–initiated lung tumors	[163]
MDSC recruitment	ZEB1 recruited MDSCs by upregulating IL6 and IL8.	Breast cancer	[162]
	Snail recruited MDSCs by upregulating CXCL1 andCXCL2.	Ovarian cancer	[174]

### Increasing Treg cells, suppressing CD8^+^ T cells

Snail, which is a typical EMT-TF, was overexpressed to generate distinct epithelial-like and mesenchymal-like melanoma tumors in vivo. Snail^high^ mesenchymal-like melanoma cells were found to be more resistant to adoptive gp70 peptide-pulsed DC transfer therapy, partially due to the induction of Tregs and immunosuppressive CD11c^+^ DCs that were activated by TGF-β and Snail-induced thrombospondin 1 (TSP1) [70]. Similarly, increased infiltration of Tregs and CD206^+^ M2 macrophages was observed in Snail^high^ mesenchymal PyMT breast cancers [84]. In addition, activation of Snail by TGF-β and IL-2 likely results in the generation of more immunosuppressive CD4^+^ CD25^-^ natural Tregs in cholangiocarcinoma with EMT-like features [161]. ZEB1, another key EMT-TF, has been reported to be directly linked with immunosuppressive effects in cancer [82, 162–164]. Using gain- and loss-of-function approaches in syngeneic mouse melanoma xenograft models, Plaschka and colleagues demonstrated that ectopic ZEB1 expression in melanoma cells is associated with reduced CD8^+^ T-cell infiltration, because it directly inhibited the secretion of T-cell-attractive chemokines, including chemokine (C-X-C motif) ligand 10 (CXCL10) [164]. The expression of indoleamine 2,3 dioxygenases (IDOs), which are key immunoregulators that dampen T-cell activation [165], is correlated with the expression of N-cadherin (a mesenchymal marker) in clinical prostate cancer. In N-cadherin^+^ regions, the IDO1 protein and its metabolite kynurenine were co-stained, with a decreased number of CD8^+^ T cells and an increased number of Tregs [166]. Additionally, chemokine (C-C motif) ligand 20 (CCL20) derived from hepatoma cells that had undergone EMT induced IDO expression in monocyte-derived macrophages, which in turn suppressed T-cell proliferation and promoted the expansion of Tregs [167].

### Promoting macrophage polarization and recruitment of M2 TAMs

Hsu et al. found that acetylation of Snail promotes the production of TNFα, chemokine (C-C motif) ligand 2 (CCL2) and chemokine (C-C motif) ligand 5 (CCL5) in cancer cells, thereby augmenting the recruitment of TAMs into the TME [168]. Interestingly, TWIST [169], miR-506-3p/FoxQ1 axis [170] and hypoxia-AXL [171] also could induce the expression of CCL2 to recruit TAMs. A recent study using K-Ras–initiated lung tumors showed that ZEB1-induced EMT is linked to immunotherapy resistance by increasing the expression of PD-L1 and CD47 by cancer cells, and then driving the polarization of adjacent TAMs into immunosuppressive M2 macrophages [163]. Besides EMT-TFs, small non-coding RNAs such as exosomal miR-106b derived from colorectal cancers with mesenchymal features induced M2 macrophage via IL-6 secretion [172], and long non-coding RNAs such as Lon-PYCR1 promotes EMT in oral squamous cell carcinoma and stimulates M2 macrophage polarization through IL-6 and VEGF-A [173].

### Increasing the recruitment of MDSC cells

Snail knockdown inhibited the growth of HM-1 mouse ovarian cancer cell in immunocompetent mice, accompanied by an increase in CD8^+^ TILs and a decrease in MDSCs, by reducing the levels of the (C-X-C Motif) chemokine receptor 2 (CXCR2) ligands, chemokine (C-X-C motif) ligand 1/2 (CXCL1/2) [174]. ZEB1 regulates the production of inflammatory cytokines in breast cancer cells, including IL-6 and IL-8, which affects the accumulation of MDSCs in vivo [162].

Other immune cells including natural killer cells were not included in this review, because we used examples that are more widely known in immune evasion via ultimately affecting CD8^+^ T cells. Also, existing checkpoint blockade therapies predominantly (re)activate tumor-specific CD8^+^ T cells. In addition to immune cells, cancer-associated fibroblasts (CAFs) have emerged as key players in interacting with EMT to affect immune response, owing to their abundance in most solid tumors and their suppressive function on immune cell infiltration, which have been well documented [175–177]. Taken together, these observations suggest that EMT-induced TFs and activated target genes regulate the immune microenvironment through multilayered mechanisms.

## Other EMP-associated changes and immune resistance

**Fig. 5:**
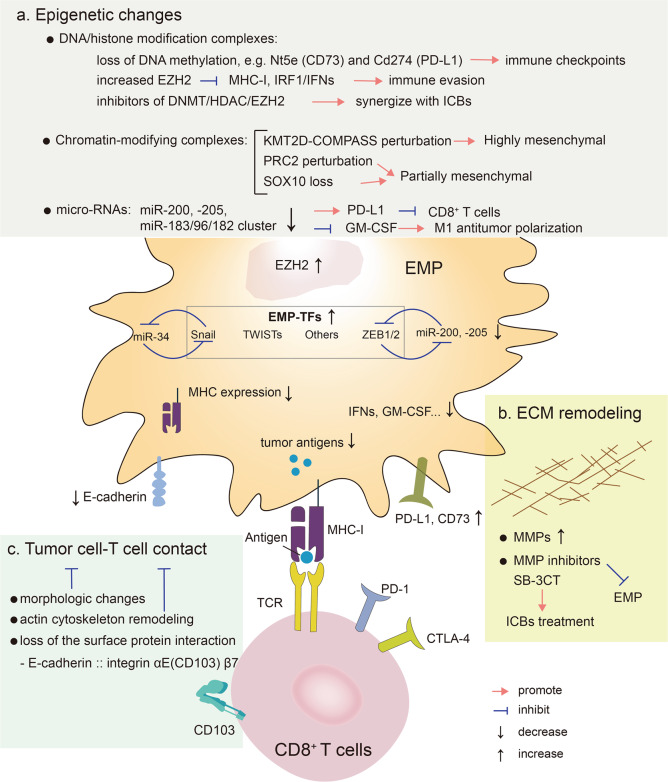
Other EMP-associated changes and immune resistance. Beyond the “classical” activation of EMT transcription factors and effectors, EMP is connected with additional pleiotropic changes, such as epigenetic alterations (**a**), ECM remodeling (**b**) and tumor cell-T cell contact (**c**). The connection between these EMP-associated changes and immune resistance is shown. Epigenetic alterations include DNA/histone modification, chromatin-modifying and microRNAs changes. DNA methylation was lost in some well-known regulators of immune evasion, including Nt5e (CD73) and Cd274 (PD-L1). Increased EZH2 levels can suppress the expression of MHC-I and mitigate IFR1 mediated IFNs signaling. Epigenetic agents, including HDAC inhibitors, DNA methyltransferase inhibitors, and EZH2 inhibitors, display promising synergies with ICBs in patients via activating immunomodulatory mechanisms, such as enhancing HLA class I antigen processing machinery (APM) component expression and function. The changes of chromatin-modifying complexes, such as KMT2D-COMPASS or PCR2 perturbation and loss of SOX10, can affect the cancer cell EMP states. Some microRNAs, including miR-200/miR-200c and microRNA-183/96/182 cluster, are repressed in cancer cells displaying EMP, which may inhibit the immune response. ECM remodeling, including increased MMPs, can stimulate EMP progression. SB-3CT, an MMP2/9 inhibitor, could improve the efficacy of anti-PD-1 and anti-CTLA-4 treatment. Morphologic changes, actin cytoskeleton remodeling and loss of the surface protein interaction can affect the cancer cell-T cell contact, resulting in different CTL-mediated killing effects. CD8 cluster of differentiation 8, COMPASS a complex of proteins associated with a trithorax-related SET domain protein, CTLA-4 cytotoxic T-lymphocyte antigen-4, EMP epithelial mesenchymal plasticity, EMP-TFs EMP inducing transcription factors, DNMT DNA methyltransferase, E-Cadherin epithelial cadherin, ECM extracellular matrix, EZH2 enhancer of zeste 2 polycomb repressive complex 2 subunit, GM-CSF granulocyte-macrophage colony-stimulating factor, HDAC histone deacetylase, KMT2D-COMPASS Histone methyltransferase complex, Nt5e ecto-5′-nucleotidase, miR micro RNA, MHC-I major histocompatibility complex I, MMPs proteases of the matrix metalloproteinases, ICB immune check point blockade, IFNs interferons, IRF1 interferon regulatory factor 1, SB-3CT a selective MMP2/9 inhibitor, Snail snail family transcriptional repressor 1, SOX10 sex-determining region Y-box 10, PD-1 programmed cell death protein 1, PD-L1 programmed death-1 ligand 1, PRC2 polycomb complex 2, TCR T cell receptor, TF transcription factor, TWISTs the basic helix–loop–helix factors, ZEB1/2 zinc finger E-Box binding homeobox factor 1/2

Beyond the “classical” activation of EMT transcription factors and effectors, EMP is connected with additional pleiotropic changes, such as epigenetic alterations, extracellular matrix (ECM) remodeling, tumor cell-T cell contact and others [178, 179]. Below we discuss their roles in EMP-mediated immune resistance (Fig. 5).

### Epigenetic alterations

The EMP plasticity is orchestrated by epigenetic alterations by signals from the microenvironment [180, 181]. A core set of EMT-TFs (SNAI1/2, TWIST1 and ZEB1/2) recruit various epigenetic regulatory complexes, notably DNA/histone modification complexes, to achieve the widespread changes in gene expression during EMT [182], by mediating a dynamic epigenetic alterations via DNA methylation and histone modifications, such as permissive H3K4me and repressive H3K27me histone marks [181]. The EMP-associated epigenetic alterations are involved in various biological process, including cell growth, cancer stemness and metastasis [181]. The mesenchymal subtype of glioblastoma multiforme stem cells (GSCs) enforced by immune attack acquires stable transcriptional and epigenetic changes, such as DNA methylation, thereby increasing immune evasion. Notably, DNA methylation was lost in some well-known regulators of immune evasion, including Nt5e (CD73) and Cd274 (PD-L1) [112]. In contrast, Wu et al. performed epigenome profiling of 60 glioblastoma primary tumors and found the chromatin modifier sex-determining region Y-box 10 (SOX10) is a master regulator in receptor tyrosine kinase I (RTK1) amplified-subtype tumors [183]. SOX10 loss causes a subtype transition to a mesenchymal cellular state via the remodeling of active enhancers, thereby increasing tumor invasion and immune cell infiltration. This leads to a reduction in the survival rate of an in vivo syngeneic graft glioblastoma mouse model. These findings suggest that the subtype transition of glioblastoma to a mesenchymal phenotype is associated with epigenetic changes but represents different outcomes of immune attack due to distinct genetic mutations or background. A genome-wide CRISPR screen identified two chromatin-modifying complexes, polycomb complex 2 (PRC2) and Histone methyltransferase KMT2D-COMPASS, which operate as critical regulators to maintain a stable epithelial state. Dysfunction of PRC2, but not KMT2D-COMPASS, was found to mediate a quasi-mesenchymal state that contributed to breast cancer metastasis [184]. The enhancer of zeste 2 polycomb repressive complex 2 subunit (EZH2), the effector subunit of the PRC2 polycomb complex, which is recruited by Snail to catalyze the H3K27me3, thereby inhibiting E-cadherin during EMT [185]. EZH2 overexpressed in a wide variety of cancers with an active EMT [186–192] and co-operated with signal transducer and activator of transcription 3 (STAT3) to regulate MHC class I antigen processing in melanoma, which mediated immune responses [193]. Furthermore, in airway fibrotic diseases, ZEB1 enhanced the catalytic activity of EZH2 in epigenetic way to silence IRF1 expression especially in mesenchymal transitioned cells, associated with inhibiting the protective mucosal interferon (IFN)-I and III production [145]. However, it is uncertain how much EZH2 contributes to EMT-mediated immune escape, as EZH2 also controls tumor growth and metastasis [189, 192, 194]. Histone deacetylases HDAC1 and HDAC2, which function as components of the Mi-2–nucleosome remodeling and deacetylase (NuRD) repressive complex, are key regulators of EMT by silencing E-cadherin [195–198]. Although epigenetic agents, including HDAC inhibitors, DNA methyltransferase (DNMT) inhibitors, and EZH2 inhibitors, display promising synergies with ICBs in patients via activating immunomodulatory mechanisms, such as enhancing HLA class I antigen processing machinery (APM) component expression and function [199–201], it is unknown how much of this benefit comes from inhibiting EMP. Thus, it would be valuable to profile the change of epigenetic regulators among tumors with different EMP states and exploit their contribution to EMP-mediated immune resistance. Besides, microRNAs regulate EMT in in a sequence-specific fashion [202]. For example, miR-200 family negatively regulates ZEB1 and ZEB2 [203–205], and deceased during EMT in cancer cells [202]. ZEB1 relieves miR-200 repression of PD-L1 on tumor cells, leading to CD8^+^ T-cell immunosuppression and metastasis [82]. Consistently, miR-200c restoration upregulates cytokines, such as granulocyte-macrophage colony-stimulating factor (GM-CSF), thereby promoting M1 antitumor macrophage polarization [206]. Another microRNA, microRNA-183/96/182 cluster (m96cl), is highly repressed in NSLC cells that have undergone EMT [207]. Ectopic expression of m96cl resulted in inhibition of migration and invasion, tumor growth and metastasis. This was found to depend on the induction of interleukin 2-mediated anti-cancer CD8^+^ cytotoxic T cell response [208].

### ECM remodeling

Integrins, an important part of ECM, are a family of ubiquitous cell member adhesion receptors, which play an essential role in several physiological processes via attachment to ECM [209]. Inhibiting the function of integrin using specific mAbs was shown to boost efficiency of ICB therapy in animal models and maintain a substantial survival benefit [210, 211]. In parallel with the launch of EMT program in cancer cells, the degradation of ECM is mediated by proteases of the matrix metalloproteinases (MMPs) family [212, 213]. Several MMPs inhibitors may function by antagonizing EMP. It has been reported that SB-3CT, an MMP2/9 inhibitor, could improve the efficacy of anti-PD-1 and anti-CTLA-4 treatment in mouse models with melanoma and lung cancer via regulating PD-L1 expression [214]. It will be of interest to study targeting EMP by anti-inflammatory compounds in the future, in particular, the development of synthetic compounds that can promote the resolution of inflammation seems to be necessary.

### Tumor cell-T cell contact

The contact of tumor cells with T cells is a potential modulator of immune response. Tumors with different degrees of EMT undergo a series of physical changes including tight junction dissolution, cell polarity alterations (e.g., Par, Crumbs, and Scribble complexes), cytoskeletal rearrangements, the loss of epithelial cell markers (e.g., E-cadherin, integrins, EpCAM, Claudin), and the emergence of mesenchymal cell markers (e.g., N-cadherin, vimentin, fibronectin) [178], which not only increase tumor motility and migration [31, 157, 195], but also affect the contact with immune cells. The disruptions of tumor cell-T cell contact and T cell receptor (TCR) signaling have been observed in vitro between CTLs and mesenchymal tumor cells [215, 216]. Acquisition of dramatic morphologic changes and actin cytoskeleton remodeling in MCF-7 human breast cancer cells is associated with an inhibition of CTL-mediated lysis, the effect of which is due to differential induction of autophagy in tumor cells [71]. Whether the effect is also affected by direct contact remains to be explored, which could be investigated by real-time imaging of cell-cell interactions. When tumor cells come into contact with T cells, molecules on the tumor cell surface may affect the activity of T cells. E-cadherin, an epithelial cell marker, interacts with integrin α_E_(CD103) β_7_, often expressed by tumor infiltrating lymphocytes (TILs), which is necessary for T cell cytolytic granule polarization and subsequent exocytosis. Either blocking CD103 with antibody or targeting E-cadherin by genetic depletion abrogated TCR-mediated cell killing [217]. Moreover, enrichment of CD103^+^ TILs is associated with improved outcomes in cancer patients and the interaction between the E-cadherin and the α_E_β_7_ integrin are important for the retention of CD8^+^ T lymphocytes in epithelial tumors [218–222]. This TIL subset displays increased cytolytic activity upon PD-1/PD-L1 blockade [219, 223], indicating that ICB therapy may be more effective in tumors with high E-cadherin expression. However, E-cadherin can also be recognized by other receptors like killer cell lectin like receptor G1 (KLRG1) on NK cells and this interaction functions as an inhibitory signal [224]. Loss of E-cadherin during EMT sensitizes tumor cells to NK-mediated cytotoxicity, suggesting that EMT confers increased susceptibility to NK cells and contributes, in part, to the inefficiency of the metastasis [225]. Once the metastases formed, T cells, not NK cells, are major sources to kill cancer cells in patients treated with ICB therapies. Therefore, investigation of the interactions between tumor surface adhesion proteins and T cells may not only enhance the antitumor activity of T cells, but also increase their infiltration, since immune cells are often excluded from the tumor core and positioned at the tumor margins.

Besides, EMP is also involved in regulating various additional aspects of tumorigenesis and metastasis, such as the regulation of stemness, cellular quiescence and escape from senescence and apoptosis [179, 226]. It is difficult to assess the specific and direct effect of EMP on immune response, as these aspects of tumors may be part of the EMP consequences, or part of the EMP activators that together with the EMP ultimately allow survival against the immune system. But EMP interconnects with ICB tumor-intrinsic or extrinsic resistance mechanisms to evade ICB therapies. When conducting in vivo/in vitro studies, researchers should consider the EMP confounding factors such as cell growth, stemness, apoptosis [87], which may primarily contribute to immune resistance.

## Potential strategies targeting EMP to enhance the ICB response

**Fig. 6:**
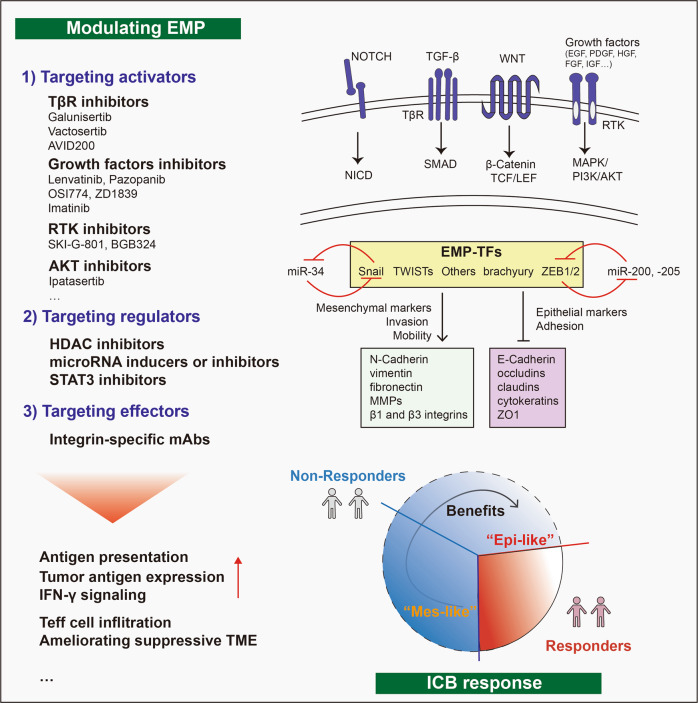
Therapeutic strategies targeting EMP to enhance ICB therapy. Strategies to modulate EMP include: 1) targeting activators, including NOTCH, TGF-β, WNT, and growth factors (e.g., EGF, PDGF, HGF, FGF and IGF signaling via RTKs); 2) targeting regulators, including EMT-TFs, such as Snail, TWISTs, brachyury and ZEB1/2, and associated miRNAs that target the above EMT-TFs; and 3) targeting effectors by inhibiting the expression/activity of factors that functionally contribute to a mesenchymal state, including N-cadherin, vimentin, fibronectin, MMP family members and β1,3 integrins, or promoting the expression/activity of factors that mediate an epithelial state, including E-cadherin, occludins, claudins, cytokeratins and ZO1. This ultimately can help improve the patient response to ICB therapy by overcoming barriers imposed by EMP, for example, by increasing the tumor antigen expression or antigen presentation and the sensitivity of IFN-γ signaling, or by ameliorating the immunosuppressive TME to increase the infiltration or function of Teff cells. AVID200 TβRII ectodomain trap, APC antigen presenting cell, EMP epithelial mesenchymal plasticity, Epi-like epithelial-like, EGF epidermal growth factor, EMP epithelial mesenchymal plasticity, FGF fibroblast growth factor, IGF insulin growth factor, HDAC histone deacetylase, HGF hepatocyte growth factor, ICB immune checkpoint blockade, IFN interferon, Mes-like mesenchymal-like, NICD NOTCH intracellular domain, PDGF platelet-derived growth factor, PI3K/AKT phosphatidylinositol-3-kinase/protein kinase B, SMAD Sma and Mad related proteins, Snail Snail family transcriptional repressor 1, STAT signal transducer and activator of transcription, TCF/LEF T-cell factor/lymphoid enhancer factor, TME tumor microenvironment, TF transcription factor, TβR TGF-β receptor, ZO1 zona occludens 1

It is well documented that various signaling pathways play a role in regulating EMP [157, 227]. Here we summarized three-level approaches to modulate EMP to overcome EMP-induced immune resistance: 1) targeting EMP activators, such as TGF-β, NOTCH, WNT, EGF or platelet derived growth factor (PDGF); 2) targeting regulators, EMT-TFs (SNAI1, SNAI2, ZEB1/2, TWIST) and others; and 3) targeting effectors, thus activating MET to reverse EMT or antagonizing the expression of chemokines and extracellular matrix (ECM) proteins (e.g., by inducing fibronectin expression) [157, 178, 228] (Fig. 6).

### Targeting EMP activators

TGF-β is a strong inducer of EMP, and its receptor signaling pathway is a promising target. Galunisertib (LY2157299), a TGF-β type I receptor (TβRI)-specific kinase inhibitor [229], was used to inhibit TGF-β receptor signaling in transplanted *Lgr5*^*eGFP-creERT2*^*::Apc*^*fl/fl*^*-Kras*^*LSL-G12D*^*-Tgfbr2*^*fl/fl*^*-Trp53*^*fl/fl*^ mouse colorectal cancer organoids (LAKTP MTOs), in the cecum of syngeneic mice [230]. This inhibitor reduced primary tumor growth and liver metastasis by blocking cancer associated fibroblast (CAF)- derived TGF-β signaling in the TME, and increasing the infiltration and function of CD8^+^ CTLs or CD4^+^ T-helper cells [230]. Interestingly, the combination of galunisertib with an anti-PD-1 or anti-PD-L1 antibody eliminated established metastases [230], as well as promoted tumor regression of a mouse xenograft models established with the Lewis lung cancer (LCC) cell line, primary esophageal squamous-cell cell line MEC2 and colorectal cancer cell line CT26 [231, 232]. Another TβRI inhibitor named Vactosertib via inhibiting ECM hyperplasia was reported to help paclitaxel to gain more easily access to tumors [233], which further may help break down the barrier between tumors and T cells. In addition, TβRII inhibitors, such as TGF-β1/3 ligand trap AVID 200, enhanced anti-tumor T cells activity via inhibiting TGF-β ligands [234].

Besides the TGF-β pathway, targeting other EMP activators (e.g., fibroblast growth factor (FGFR), EGFR, AXL) is also promising. For example, targeting FGFR using lentivatinib aids to improve anti-PD-1 efficiency via reducing tumor PD-L1 level and Treg differentiation [235], remodeling immunosuppressive TME and increasing functional CD8^+^ T cells [236]. Targeting EGFR using either OSI774 [237] or ZD1839 [238] enhances the efficacy of PD-1 blockade. Another target, tyrosine receptor kinase AXL, has been found aberrantly activated in several cancer types [239–241] and is also an essential EMT inducer [241–243]. AXL and its ligand, i.e., growth arrest-specific 6 (GAS6) proteins axis is reported to promote cancer cell proliferation, survival, migration, invasion, and immune evasion [244]. It’s well documented that AXL functions in triggering an immunosuppressive tumor microenvironment resulting in immune evasion [245]. In a hypoxia-induced tumor plasticity model, human lung cancer clones with mesenchymal features were more resistant to NK- and CTL-mediated cytotoxicity than clones with epithelial features. A small-molecule, bemcentinib (also known as BGB324), targeting AXL re-sensitizes mesenchymal lung cancer cells to cell cytotoxicity [246]. The AXL targeting agents in preclinical and clinical development include selective antibodies (e.g., YW327.6S2, D9, E8), selective small-molecule inhibitors (e.g., R428, DP3975) and more nonselective tyrosine receptor kinase inhibitors (e.g., BMS-777607, Cabozantinib, Gilteritinib, Sitravatinib, Crizotinib) [244, 245, 247]. In vivo pharmacologic inhibiting pan-TAM Tyrosine Kinases, TYRO3, AXL, and MERTK, diminishes MDSC suppressive capability, promotes CD8^+^ T cell infiltration, slows tumor growth, and augments anti-PD-1 immunotherapy [247–249]. Consistently, another AXL kinase inhibitor, SKI-G-801, blocks metastasis through inducing anti-tumor immune responses and potentiates anti-PD-1 therapy in multiple mouse cancer models (including B16F10 melanoma, CT26 colon, 4T1 breast, TC1 and C3PQ lung cancer models) [250, 251]. Similarly, combining AXL kinase inhibitor R428 with anti–PD-1 in a mouse HER2^+^ breast cancer model reduces the primary tumor and metastatic burdens [171]. An AXL-targeting antibody–drug, Enapotamab vedotin (EnaV), effectively enhances ICB benefit in human BLM melanoma and lung LCLC-103H cancer models [252].These results unveiled a potentially promising combination therapy, which synergistically targets EMP via extracellular inducers and their transmembrane receptors.

Targeting intracellular transducers of EMP, such as AMP-activated protein kinase (AMPK) or AKT serine/threonine protein kinase, is another strategy to block the signaling transduction of EMP. As these transducers are involved in multiple signaling pathways, as well as multiple biological processes, the efficiency and specificity should be carefully considered when targeting them. Metformin, a widely used antidiabetic agent was found to reverse EMT in EGFR-tyrosine kinase inhibitor (TKI)-resistant human lung cancers [253]. The combination of metformin with the protein kinase inhibitor, sorafenib, suppressed EMT and significantly minimized the postoperative recurrence and lung metastasis of hepatocellular carcinoma in an orthotopic mouse model [254], and induced coiled-coil domain containing 65 (CCDC65) expression to suppress alpha-enolase (ENO1)-AKT1 pathway-mediated EMT in gastric cancer cells [255]. Metformin functions as an activator of AMPK to reverse the mesenchymal phenotype of cancer cells by targeting the AKT-E3 ubiquitin ligase MDM2-FOXO3a axis in 4T1 breast cancer and PC-3 prostate cancer cells [256]. In addition to effectively targeting EMP to reduce tumor progression, metformin also contributes to ameliorating the immunosuppressive TME. Metformin has been reported to increase CTL activity by reducing the stability and membrane localization of PD-L1 [257]; more specifically, metformin was found to decrease the cellular abundance of PD-L1 by disrupting the electrostatic interaction between the PD-L1 cytoplasmic domain and cell membranes [258]. In addition, metformin enhance the efficacy of PD-1 blockade by reducing intra-tumoral hypoxia [259] or by stimulating the production of mitochondrial reactive oxygen species (mtROS) to ensure CD8^+^ CTL proliferation [260]. In addition, combining metformin with CTLA-4 blockade also helped to increase the efficacy of immunotherapy [257, 261].

### Targeting EMP regulators

Besides SNAI, ZEB and TWIST families, which are 3 famous transcriptional factors contributing to promote EMT, STAT3 pathways and epigenetic regulation such as HDAC are also critical to this process.

STAT3 can induce EMT [262, 263], and STAT3 inhibitors have been shown to inhibit EMT in different systems [264–266]. Of particular interest is the behavior of napabucasin, a small molecule inhibitor of STAT3, which has been in active clinical development (up to Phase II) for the treatment of metastatic colorectal cancer, and helps to enhance the response to anti-PD-1 therapy [267].

Due to difficulties in targeting EMT-TFs directly, another promising approach is to apply modified synthetic miRNAs which interfere with EMT-TFs at the posttranscriptional level. In this respect, miR-34a is a good example, which mediates the suppression of EMT by directly inhibiting Snail expression [268, 269]. MRX34 is a therapeutic liposome that was used to deliver miR-34a mimics into a syngeneic mouse lung adenocarcinoma model, promoting the recruitment of TILs and reducing the population of CD8^+^ PD-1^+^ exhausted T cells in vivo via the p53/miR-34/PD-L1 axis [270]. Regarding EMT-favoring compounds that act on epigenetic changes, several histone deacetylase (HDAC) inhibitors have been shown to inhibit EMT [271, 272]. Importantly, the effects of HDAC inhibitors [273, 274] also augmented the therapeutic effect of anti-PD-1 therapy.

### Targeting EMP effectors

Another therapeutic approach is to target the interaction between cancer cells and ECM. Integrins are a family of ubiquitous cell member adhesion receptors, which play an essential role in several physiological processes via attachment to ECM [275]. Integrins are an attractive target due to their crucial role in tumor progress and metastasis. Inhibiting the function of integrin using specific mAbs was shown to boost the efficiency of ICB therapy in animal models and maintain a substantial survival benefit [210, 211]. The degradation of ECM which is needed for cancer cell invasion, is mediated by proteases of the MMPs family. MMPs inhibitors may function (at least in part) by inhibiting EMP. It has been reported that SB-3CT, an MMP2/9 inhibitor, could improve the efficacy of anti-PD-1 and anti-CTLA-4 treatment in mouse models with melanoma and lung cancer via regulating PD-L1 expression [276].

In Table 2, we list additional molecules and agents regulate EMP to boost immunotherapy. It should be pointed out that further in-depth mechanistic research on EMP-targeting drugs is needed before clinical translation can be initiated. More insights are needed as to how these types of drugs can best be combined with ICB for each cancer patient to achieve the optimal response. For example, in an autochthonous BRAF^V600E^PTEN^-/-^ melanoma model, a TβRI kinase inhibitor augmented the effects of anti-CTLA-4 treatment but failed to augment the effects of anti-PD-1/PD-L1 blockade. Mechanistically, the TβRI kinase inhibitor was found to stimulate the cleavage of MMP9-dependent PD-L1, which led to resistance to anti-PD-1 therapy [277]. Therefore, to maximize future prospects and directions, it is important to understand the precise mechanism of action of the drug and its effects on the TME, and then design the appropriate combination of treatment in a patient tailored manner. Targeting EMP by anti-inflammatory compounds will be an interesting topic for future studies, in particular, the development of synthetic compounds that can promote the resolution of inflammation seems to be promising.

**Table 2:** Strategies for targeting EMP

Targeting EMP	Mouse model	How	Reference
**Targeting extracellular inducers and receptors**			
**TGFβ-TβRI inhibitors**			
Galunisertib/ LY2157299 (TβRI inhibitor)	Colon cancer	Maintained the cytotoxic T-cell response and rendered tumors susceptible to anti-PD-1/PD-L1 therapy.	[230]
	NSCLC and esophageal squamous-cell carcinoma (ESCC)	Promoted T-cell infiltration from the stroma into the tumor, enhanced the response to anti–PD-1 therapy.	[232]
	Melanoma	Enhanced the CTL response via ubiquitin-mediated degradation of Smad4.	[289]
	TNBC, 4T1 model	Increased T-cell numbers in treated tumors.	[231]
Vactosertib (TβRI inhibitor)	Orthotopic pancreatic cancer models	Inhibited ECM hyperplasia to allow paclitaxel to more easily access cancer cells.	[233]
AVID200 (selective inhibitor of TGF-β 1&3)	TNBC, 4T1 model	Enhanced anti-tumor T-cell activity.	[234]
**FGFR inhibitors** (tyrosine kinase inhibitors that non-specifically target FGFR)			
Lenvatinib	Hepatocellular carcinoma	Reduced the tumor PD-L1 level and Treg differentiation to improve anti-PD-1 efficacy by blocking FGFR4.	[235]
	Renal cell carcinoma (RCC) cell lines	Decreased the population of TAMs and increased that of IFN-γ^+^ CD8^+^ T cells.	[236]
Pazopanib	Metastatic RCC	Inhibited the ERK/β-CATENIN pathway to prime DCs.	[290]
EGFR inhibitors			
OSI774	EGFR-mutated NSCLC	Decreased the number of CD4^+^ effector regulatory T cells, and infiltration into the TME and enhanced the efficacy of anti-PD-1 mAb therapy.	[237]
ZD1839	Syngeneic mouse models	Destabilized PD-L1 and enhanced the therapeutic efficacy of PD-1 blockade.	[238]
**RTK inhibitors (eg. Targeting AXL)**			
Cabozantinib	Advanced kidney cancer	Improved progression-free survival and the OS benefit when combined with nivolumab.	[291]
	metastatic castration-resistant prostate cancer (mCRPC)	Targeted mCRPC-infiltrating MDSCs and demonstrated a synergistic therapeutic response with ICB.	[292]
RXDX-106 (pan-TAM- TYRO3, AXL, MER small-molecule kinase inhibitor)	Multiple syngeneic mouse models	Increased intratumoral CD8^+^ T cells and potentiated the effects of α-PD-1 Ab treatment.	[247]
UNC4241	Melanoma	Increased CD8+ T-cell infiltration, and augmented anti–PD-1 checkpoint inhibitor immunotherapy.	[248]
BGB324 (Bemcentinib)	EGFR mutation-positive lung adenocarcinomas	Decreased the PD-L1 and CXCR6 mRNA levels.	[293]
	Mesenchymal-like glioblastoma tumors	Co-treatment with anti-BGB324 and anti-PD-1 antibodies improved survival in mouse GBM models.	[294]
	Lung cancers	Sensitized mesenchymal lung cancer cells to CTLs and NK cells via intracellular adhesion molecule-1 (ICAM-1)/leukocyte function-associated antigen-1 (ICAM1/LFA-1) and UL16 binding protein 1 (ULBP1)/ natural killer group 2, member D (NKG2D) interactions.	[246]
BMS-777607	Murine Model of Triple-Negative Breast Cancer	Blocks macrophage efferocytosis and Gas6-PS–opsonized apoptotic cell, and enhances anti-PD-1 mAb efficacy via up-regulating PD-L1 expression	[249]
SKI-G-801	B16F10 melanoma, CT26 colon and 4T1 breast model	Blocks metastasis through inducing CD8^+^ T cells, decreasing M2 macrophage and potentiates anti-PD-1 therapy	[250]
	TC1 and C3PQ mouse tumor models	Improves efficiency of anti-PD-1 therapy, exhibiting increased proportion of effector memory helper T cells, CD86+ macrophages.	[251]
Enapotamab vedotin (EnaV)	Melanoma and lung cancer models	Induced ICB benefit and promoted the induction of a memory-like phenotype in cytotoxic T cells.	[252]
R428	HER2^+^ breast cancer	Enhances anti-PD-1 responses via increased CD8^+^ T cells	[171]
**PDGFR inhibitors**			
Imatinib	Gastrointestinal stromal tumors	Abrogated the IFN-γ induced upregulation of PD-L1 via STAT1 inhibition.	[295]
**MET inhibitors**			
Crizotinib	NSCLC	In combination with cisplatin, induced immunogenic cell death by increasing PD-1 and PD-L1 levels in tumors and increased the response to anti-PD-1 treatment.	[296]
	ALK positive anaplastic lymphomas (ALCLs)	Decreased the PD-L1 level and promoted HLA class I antigen presentation.	[297]
**Targeting intracellular transducers**			
**AMPK activators**			
Metformin	Breast, melanoma and colorectal cancer (CRC) models (4T1-Luc2, B16-F10, CT26)	By reducing the stability and membrane localization of PD-L1, CTL activity was increased. Metformin boosted the efficacy of CTLA-4 immunotherapy.	[257]
	Hepatoma and TNBC models (H22 tumor-bearing mice, 4T1)	Repolarized M2-like TAMs to M1-like phenotype, resulting in the recruitment of CD8^+^ T cells and an improved therapeutic effect of anti-PD-1 antibody therapy.	[298]
**PI3K/AKT/mTOR inhibitors**			
Ipatasertib	Multiple tumor xenograft models	Blocked AKT signaling in vivo and resulted in potent antitumor activity.	[299]
**Targeting regulators**			
**HDAC inhibitors**			
Vorinostat (SAHA)	Melanoma xenografts	Promoted SOX2 degradation and augmented the therapeutic effect of anti-PD-1 therapy.	[273]
Romidepsin	Lung adenocarcinoma mouse models	Increased chemokine expression, enhanced T-cell infiltration and enhanced the response to PD-1 blockade immunotherapy.	[274]
Panobinostat	Patients with Hodgkin lymphoma	Inhibited PD-1 expression in T cells.	[300]
**MiRNA inducers or inhibitors**			
MRX34 (Snail inhibitor)	NSCLC	Promoted TILs and reduced CD8^+^ PD-1^+^ cells in vivo via the p53/miR-34/PD-L1 axis.	[270]
**STAT3 inhibitors**			
Napabucasin (BBI608)	Microsatellite-stable colorectal cancer	Enhanced the response to anti-PD-1 therapy.	[267]
**Targeting downstream effectors**			
**Adhesion molecule inhibitors**			
Integrin-specific mAbs	Metastatic TNBC models	Integrin αvβ6/8 mAb induced a substantial survival benefit in combination with anti-PD-1 therapy.	[210]
	Metastatic pulmonary melanoma and osteosarcoma models	In vitro-generated CD103^+^ conventional DCs enhanced the response to CTLA-4 therapy.	[211]

Above all, due to the plasticity and heterogeneity of various pathways functioned in EMT, development of clinical drugs to target EMT inducers, regulators, or effectors is challenging but meaningful for boosting efficiency of ICB therapy in the near future.

## Combination therapy with ICB and EMP-targeted agents in clinical trials

In Table 3, we list recent clinical trials of EMP-targeted drugs in combination with ICBs, which may facilitate the response to immunotherapy. For example, bintrafusp α (a bifunctional fusion protein) targets TGF-β and PD-L1 [278]. Vactosertib is a highly potent small molecule TGFβRI kinase inhibitor [178], and NIS793 inhibits TGF-β [279]. GT90001 is an anti-activin receptor-like kinase-1 (ALK-1) antibody [280]. Sitravatinib and lenvatinib target intracellular signaling kinases [281, 282], and are combined with anti-PD-1/PD-L1 or anti-CTLA-4 treatment in different cancer types. These combination trials are remarkable advances, although most of the trials are still ongoing.

**Table 3:** Clinical trials of EMP-targeted agents in combination with ICBs

EMP-targeted agents	ICBs	Cancer types	Clinical trial status (phase)	Clinical trial number (study start)
Bintrafusp alfa (anti-PD-1/PD-L1 and TβRII-Fc fusion protein)	Bintrafusp alfa, Pembrolizumab (anti-PD-1 antibody)	NSCLC	Active, not recruiting (III)	NCT03631706 (Oct 19^th^, 2018)
	Bintrafusp alfa, Durvalumab (anti-PD-L1 antibody)	NSCLC	Active, not recruiting (II)	NCT03840902 (April 16^th^, 2019)
Vactosertib (TβRI kinase inhibitor)	Pembrolizumab (anti-PD-1 antibody)	PD-L1 positive NSCLC	Recruiting (II)	NCT04515979 (Dec 17^th^, 2020)
	Durvalumab (anti-PD-L1 antibody)	Urothelial carcinoma	Not yet recruiting (II)	NCT04064190 (Oct 15^th^, 2021)
	Durvalumab (anti-PD-L1 antibody)	Gastric cancer	Not yet recruiting (II)	NCT04893252 (June 1^st^, 2021)
NIS793 (anti-TGF-β antibody)	Spartalizumab (anti-PD-1 antibody)	Metastatic pancreatic ductal adenocarcinoma	Recruiting (II)	NCT04390763 (Oct 16^th^, 2020)
GT90001(ALK-1 antibody)	Nivolumab (anti-PD-1 antibody)	Hepatocellular carcinoma	Active, not recruiting (I, II)	NCT03893695 (May 25^th^, 2019)
	KN046 (anti-PD-1/CTLA-4 antibody)	Solid tumors	Recruiting (I, II)	NCT04984668 (Nov 2^nd^, 2021)
Lenvatinib (multiple kinase inhibitor: EGFR1/VEGFR/ VEGFR3)	Pembrolizumab (anti-PD-1 antibody)	Head and neck squamous cell carcinoma	Recruiting (III)	NCT04199104 (Feb 5^th^, 2020)
Sitravatinib (receptor tyrosine kinase inhibitor: TYRO3/AXL/MERTK)	Pembrolizumab (anti-PD-1 antibody)	Lung cancers	Not yet recruiting (II)	NCT04925986 (Jan 1^st^, 2022)
	Pembrolizumab or Nivolumab (anti-PD-1 antibody)	Advanced or metastatic solid malignancies	Recruiting (II, III)	NCT04887870 (June 30^th^, 2021)
	Nivolumab (anti-PD-1 antibody)	Clear cell renal cell carcinoma	Active, not recruiting (II)	NCT03680521 (Oct 10^th^, 2018)

Active, not recruiting: The study is ongoing, and participants are receiving an intervention or being examined, but potential participants are not currently being recruited or enrolled

Phase I: dose-ranging on healthy volunteers for safety

Phase II: testing of drug on participants to assess efficacy and side effects

Phase III: testing of drug on participants to assess efficacy, effectiveness and safety

## Concluding remarks

Accumulating evidence has demonstrated tumor EMP is a clinically relevant mechanism for immune evasion [56–61]. The observation that non-responders to ICB therapy are more likely to exhibit expression of mesenchymal markers [98–100], suggests that EMP targeting may boost ICB therapy. Moreover, by applying different methods to investigate the interplay between cancer cell EMP and ICB response, it’s becoming increasingly clear that EMP renders tumors more resistant to ICB therapies. The traditional in vitro or in vivo models provide methods to mimic simple states of EMP and investigate their impact on immunotherapy resistance, while the application of more precise and comprehensive technical advances such as single-cell sequencing and dual-reporters lineage tracing systems provide new insights and clarify for example which dynamic changes in specific cell populations contribute to immunotherapy resistance.

In this review, we summarized the mechanism of how EMP regulates immunotherapy resistance from 3 aspects: EMP mediated tumor-intrinsic, -extrinsic mechanisms and other EMP-associated changes. EMP displays multilayered changes to directly affect CD8^+^ T mediated killing, such as decreased tumor antigen, antigen presentation machinery, deficiency of IFN-γ signaling and increased immune checkpoints or indirectly by modifying the immunosuppressive microenvironment. It remains unclear which effectors of EMP play a major role in different cancer types and whether there are biomarkers of EMP that can predict tumor sensitivity to ICB therapy.

Since EMP plays multidimensional roles in tumor development [178, 179], it’s possible that targeting or reversing EMP will alleviate/overcome multiple resistance mechanisms. Disrupting tumor-intrinsic resistance, and reshaping the immune microenvironment to reinvigorate anti-tumor T cells are the main avenues to improve ICB therapy response in cancer patients [283]. We discussed the current strategies to modulating tumor EMP signaling and the potential of combining ICB therapy and EMP targeting therapeutic approaches to enhance anti-tumor efficiency of T cells in different cancer types [284]. Remarkable progress has been made in pre-clinical studies. Most of the combination trials are still in progress and the results are eagerly awaited. At the same time, the potential toxicity or side effects of EMP-targeting drugs need to be considered and carefully monitored.

## References

[1] Wei SC, Duffy CR, Allison JP (2018). Fundamental Mechanisms of Immune Checkpoint Blockade Therapy. Cancer Discov.

[2] Hodi FS, O’Day SJ, McDermott DF, Weber RW, Sosman JA, Haanen JB (2010). Improved Survival with Ipilimumab in Patients with Metastatic Melanoma. N. Engl J Med.

[3] Weber J, Mandala M, Del Vecchio M, Gogas HJ, Arance AM, Cowey CL (2017). Adjuvant Nivolumab versus Ipilimumab in Resected Stage III or IV Melanoma. N. Engl J Med.

[4] Larkin J, Chiarion-Sileni V, Gonzalez R, Grob J-J, Rutkowski P, Lao CD (2019). Five-Year Survival with Combined Nivolumab and Ipilimumab in Advanced Melanoma. N. Engl J Med.

[5] Cascone T, William WN, Weissferdt A, Leung CH, Lin HY, Pataer A (2021). Neoadjuvant nivolumab or nivolumab plus ipilimumab in operable non-small cell lung cancer: the phase 2 randomized NEOSTAR trial. Nat Med.

[6] Kwapisz D (2021). Pembrolizumab and atezolizumab in triple-negative breast cancer. Cancer Immunol Immunother.

[7] Bagchi S, Yuan R, Engleman EG (2021). Immune Checkpoint Inhibitors for the Treatment of Cancer: Clinical Impact and Mechanisms of Response and Resistance. Annu Rev Pathol.

[8] Ribas A, Wolchok JD (2018). Cancer immunotherapy using checkpoint blockade. Science.

[9] Twomey JD, Zhang B (2021). Cancer Immunotherapy Update: FDA-Approved Checkpoint Inhibitors and Companion Diagnostics. AAPS J.

[10] Tawbi HA, Schadendorf D, Lipson EJ, Ascierto PA, Matamala L, Castillo Gutiérrez E (2022). Relatlimab and Nivolumab versus Nivolumab in Untreated Advanced Melanoma. N. Engl J Med.

[11] Boumber Y (2018). Tumor mutational burden (TMB) as a biomarker of response to immunotherapy in small cell lung cancer. J Thorac Dis.

[12] Gong J, Chehrazi-Raffle A, Reddi S, Salgia R (2018). Development of PD-1 and PD-L1 inhibitors as a form of cancer immunotherapy: a comprehensive review of registration trials and future considerations. J Immunother Cancer.

[13] Wang S, He Z, Wang X, Li H, Liu X-S (2019). Antigen presentation and tumor immunogenicity in cancer immunotherapy response prediction. eLife.

[14] Sharma P, Hu-Lieskovan S, Wargo JA, Ribas A (2017). Primary, Adaptive, and Acquired Resistance to Cancer Immunotherapy. Cell.

[15] Schoenfeld AJ, Hellmann MD (2020). Acquired Resistance to Immune Checkpoint Inhibitors. Cancer Cell.

[16] Fares CM, Van Allen EM, Drake CG, Allison JP, Hu-Lieskovan S (2019). Mechanisms of Resistance to Immune Checkpoint Blockade: Why Does Checkpoint Inhibitor Immunotherapy Not Work for All Patients?. Am Soc Clin Oncol Educ Book.

[17] Kalaora S, Nagler A, Wargo JA, Samuels Y. Mechanisms of immune activation and regulation: lessons from melanoma. Nat Rev Cancer. 2022;22:195–207. 10.1038/s41568-022-00442-9 35105962

[18] Vesely MD, Zhang T, Chen L (2022). Resistance Mechanisms to Anti-PD Cancer Immunotherapy. Annu Rev Immunol.

[19] Tian X-J, Zhang H, Xing J (2013). Coupled Reversible and Irreversible Bistable Switches Underlying TGFβ-induced Epithelial to Mesenchymal Transition. Biophys J.

[20] Williams ED, Gao D, Redfern A, Thompson EW (2019). Controversies around epithelial–mesenchymal plasticity in cancer metastasis. Nat Rev Cancer.

[21] Yang J, Antin P, Berx G, Blanpain C, Brabletz T, Bronner M (2020). Guidelines and definitions for research on epithelial–mesenchymal transition. Nat Rev Mol Cell Biol.

[22] Bakir B, Chiarella AM, Pitarresi JR, Rustgi AK (2020). EMT, MET, Plasticity, and Tumor Metastasis. Trends Cell Biol.

[23] Ye X, Weinberg RA (2015). Epithelial–Mesenchymal Plasticity: A Central Regulator of Cancer Progression. Trends Cell Biol.

[24] Lu W, Kang Y (2019). Epithelial-Mesenchymal Plasticity in Cancer Progression and Metastasis. Dev Cell.

[25] da Silva-Diz V, Lorenzo-Sanz L, Bernat-Peguera A, Lopez-Cerda M, Muñoz P (2018). Cancer cell plasticity: Impact on tumor progression and therapy response. Semin Cancer Biol.

[26] Zhuang X, Zhang H, Hu G (2019). Cancer and Microenvironment Plasticity: Double-Edged Swords in Metastasis. Trends Pharm Sci.

[27] Terry S, Savagner P, Ortiz-Cuaran S, Mahjoubi L, Saintigny P, Thiery J-P (2017). New insights into the role of EMT in tumor immune escape. Mol Oncol.

[28] Dongre A, Weinberg RA (2019). New insights into the mechanisms of epithelial–mesenchymal transition and implications for cancer. Nat Rev Mol Cell Biol.

[29] Soundararajan R, Fradette JJ, Konen JM, Moulder S, Zhang X, Gibbons DL (2019). Targeting the Interplay between Epithelial-to-Mesenchymal-Transition and the Immune System for Effective Immunotherapy. Cancers.

[30] Horn LA, Fousek K, Palena C (2020). Tumor Plasticity and Resistance to Immunotherapy. Trends Cancer.

[31] Mullins R, Pal A, Barrett TF, Heft Neal ME, Puram SV. Epithelial-mesenchymal plasticity in tumor immune evasion. Cancer Res. 2022;82:2329–43. 10.1158/0008-5472.CAN-21-4370 PMC9256788 35363853

[32] Sexén L (1970). Epithelial-mesenchymal interactions: 18th Hahnemann Symposium. Teratology.

[33] Potts JD, Runyan RB (1989). Epithelial-mesenchymal cell transformation in the embryonic heart can be mediated, in part, by transforming growth factor beta. Dev Biol.

[34] Leptin M (1991). twist and snail as positive and negative regulators during Drosophila mesoderm development. Genes Dev.

[35] Nieto MA, Sargent MG, Wilkinson DG, Cooke J (1994). Control of cell behavior during vertebrate development by Slug, a zinc finger gene. Science.

[36] Savagner P, Yamada KM, Thiery JP (1997). The zinc-finger protein slug causes desmosome dissociation, an initial and necessary step for growth factor-induced epithelial-mesenchymal transition. J Cell Biol.

[37] Cano A, Pérez-Moreno MA, Rodrigo I, Locascio A, Blanco MJ, del Barrio MG (2000). The transcription factor snail controls epithelial-mesenchymal transitions by repressing E-cadherin expression. Nat Cell Biol.

[38] Iwano M, Plieth D, Danoff TM, Xue C, Okada H, Neilson EG (2002). Evidence that fibroblasts derive from epithelium during tissue fibrosis. J Clin Investig.

[39] Lee M, Vasioukhin V (2008). Cell polarity and cancer-cell and tissue polarity as a non-canonical tumor suppressor. J Cell Sci.

[40] Meulmeester E, ten Dijke P (2010). Integration of transcriptional signals at the tumor cell invasive front. Cell Cycle Georget Tex.

[41] Mittal V. Epithelial Mesenchymal Transition in Tumor Metastasis. Annu Rev Pathol. 2018;13:395–412. 10.1146/annurev-pathol-020117-043854 29414248

[42] Kröger C, Afeyan A, Mraz J, Eaton EN, Reinhardt F, Khodor YL (2019). Acquisition of a hybrid E/M state is essential for tumorigenicity of basal breast cancer cells. Proc Natl Acad Sci.

[43] Pastushenko I, Brisebarre A, Sifrim A, Fioramonti M, Revenco T, Boumahdi S (2018). Identification of the tumour transition states occurring during EMT. Nature.

[44] Pastushenko I, Blanpain C (2019). EMT Transition States during Tumor Progression and Metastasis. Trends Cell Biol.

[45] Bierie B, Pierce SE, Kroeger C, Stover DG, Pattabiraman DR, Thiru P (2017). Integrin-β4 identifies cancer stem cell-enriched populations of partially mesenchymal carcinoma cells. Proc Natl Acad Sci.

[46] Simeonov KP, Byrns CN, Clark ML, Norgard RJ, Martin B, Stanger BZ (2021). Single-cell lineage tracing of metastatic cancer reveals selection of hybrid EMT states. Cancer Cell.

[47] Schliekelman MJ, Taguchi A, Zhu J, Dai X, Rodriguez J, Celiktas M (2015). Molecular Portraits of Epithelial, Mesenchymal, and Hybrid States in Lung Adenocarcinoma and Their Relevance to Survival. Cancer Res.

[48] Pastushenko I, Brisebarre A, Sifrim A, Fioramonti M, Revenco T, Boumahdi S (2018). Identification of the tumour transition states occurring during EMT. Nature.

[49] Pastushenko I, Mauri F, Song Y, de Cock F, Meeusen B, Swedlund B, et al. Fat1 deletion promotes hybrid EMT state, tumour stemness and metastasis. Nature. 2021;589:448–55. 10.1038/s41586-020-03046-1 PMC7612440 33328637

[50] Pastushenko I, Blanpain C (2019). EMT Transition States during Tumor Progression and Metastasis. Trends Cell Biol.

[51] McFaline-Figueroa JL, Hill AJ, Qiu X, Jackson D, Shendure J, Trapnell C (2019). A pooled single-cell genetic screen identifies regulatory checkpoints in the continuum of the epithelial-to-mesenchymal transition. Nat Genet.

[52] Nieto MA, Huang RY-J, Jackson RA, Thiery JP (2016). EMT: 2016. Cell.

[53] Friedl P, Alexander S (2011). Cancer Invasion and the Microenvironment: Plasticity and Reciprocity. Cell.

[54] McGranahan N, Swanton C (2017). Cancer Evolution Constrained by the Immune Microenvironment. Cell.

[55] Yang D. Lineage tracing reveals the phylodynamics, plasticity, and paths of tumor evolution. Cell. 2022;45. 10.1016/j.cell.2022.04.015 PMC9452598 35523183

[56] Yu M, Bardia A, Wittner BS, Stott SL, Smas ME, Ting DT (2013). Circulating Breast Tumor Cells Exhibit Dynamic Changes in Epithelial and Mesenchymal Composition. Science.

[57] Satelli A, Mitra A, Brownlee Z, Xia X, Bellister S, Overman MJ (2015). Epithelial–Mesenchymal Transitioned Circulating Tumor Cells Capture for Detecting Tumor Progression. Clin Cancer Res.

[58] Polioudaki H, Agelaki S, Chiotaki R, Politaki E, Mavroudis D, Matikas A (2015). Variable expression levels of keratin and vimentin reveal differential EMT status of circulating tumor cells and correlation with clinical characteristics and outcome of patients with metastatic breast cancer. BMC Cancer.

[59] Wu S, Liu S, Liu Z, Huang J, Pu X, Li J (2015). Classification of Circulating Tumor Cells by Epithelial-Mesenchymal Transition Markers. PLOS ONE.

[60] Hyun K-A, Koo G-B, Han H, Sohn J, Choi W, Kim S-I (2016). Epithelial-to-mesenchymal transition leads to loss of EpCAM and different physical properties in circulating tumor cells from metastatic breast cancer. Oncotarget.

[61] Boral D, Vishnoi M, Liu HN, Yin W, Sprouse ML, Scamardo A (2017). Molecular characterization of breast cancer CTCs associated with brain metastasis. Nat Commun.

[62] Li Y, Lv Z, Zhang S, Wang Z, He L, Tang M (2020). Genetic Fate Mapping of Transient Cell Fate Reveals N-Cadherin Activity and Function in Tumor Metastasis. Dev Cell.

[63] Lüönd F, Sugiyama N, Bill R, Bornes L, Hager C, Tang F (2021). Distinct contributions of partial and full EMT to breast cancer malignancy. Dev Cell.

[64] Blank CU, Haanen JB, Ribas A, Schumacher TN (2016). The “cancer immunogram”. Science.

[65] Patel SJ, Sanjana NE, Kishton RJ, Eidizadeh A, Vodnala SK, Cam M (2017). Identification of essential genes for cancer immunotherapy. Nature.

[66] Dijkstra KK, Cattaneo CM, Weeber F, Chalabi M, van de Haar J, Fanchi LF (2018). Generation of Tumor-Reactive T Cells by Co-culture of Peripheral Blood Lymphocytes and Tumor Organoids. Cell.

[67] Cattaneo CM (2020). Tumor organoid–T-cell coculture systems. Nat Protoc.

[68] Cózar B, Greppi M, Carpentier S, Narni-Mancinelli E, Chiossone L, Vivier E (2021). Tumor-Infiltrating Natural Killer Cells. Cancer Discov.

[69] Voabil P, de Bruijn M, Roelofsen LM, Hendriks SH, Brokamp S, van den Braber M (2021). An ex vivo tumor fragment platform to dissect response to PD-1 blockade in cancer. Nat Med.

[70] Kudo-Saito C, Shirako H, Takeuchi T, Kawakami Y (2009). Cancer metastasis is accelerated through immunosuppression during Snail-induced EMT of cancer cells. Cancer Cell.

[71] Akalay I, Janji B, Hasmim M, Noman MZ, André F, De Cremoux P (2013). Epithelial-to-Mesenchymal Transition and Autophagy Induction in Breast Carcinoma Promote Escape from T-cell–Mediated Lysis. Cancer Res.

[72] Chen X-H, Liu Z-C, Zhang G, Wei W, Wang X-X, Wang H (2015). TGF-β and EGF induced HLA-I downregulation is associated with epithelial-mesenchymal transition (EMT) through upregulation of snail in prostate cancer cells. Mol Immunol.

[73] David JM, Hamilton DH, Palena C. MUC1 upregulation promotes immune resistance in tumor cells undergoing brachyury-mediated epithelial-mesenchymal transition. Oncoimmunology. 2016;5:e1117738. 10.1080/2162402X.2015.1117738 PMC4839328 27141403

[74] Hamilton DH, Huang B, Fernando RI, Tsang K-Y, Palena C (2014). WEE1 Inhibition Alleviates Resistance to Immune Attack of Tumor Cells Undergoing Epithelial–Mesenchymal Transition. Cancer Res.

[75] Hamilton DH, Griner LM, Keller JM, Hu X, Southall N, Marugan J (2016). Targeting Estrogen Receptor Signaling with Fulvestrant Enhances Immune and Chemotherapy-Mediated Cytotoxicity of Human Lung Cancer. Clin Cancer Res.

[76] Taki M, Abiko K, Ukita M, Murakami R, Yamanoi K, Yamaguchi K (2021). Tumor Immune Microenvironment during Epithelial–Mesenchymal Transition. Clin Cancer Res.

[77] López-Soto A, Huergo-Zapico L, Galván JA, Rodrigo L, de Herreros AG, Astudillo A, et al. Epithelial–Mesenchymal Transition Induces an Antitumor Immune Response Mediated by NKG2D Receptor. J Immunol. 2013;190:4408–19. 10.4049/jimmunol.1202950 23509364

[78] Rhim AD, Mirek ET, Aiello NM, Maitra A, Bailey JM, McAllister F (2012). EMT and Dissemination Precede Pancreatic Tumor Formation. Cell.

[79] Aiello NM, Maddipati R, Norgard RJ, Balli D, Li J, Yuan S (2018). EMT Subtype Influences Epithelial Plasticity and Mode of Cell Migration. Dev Cell.

[80] Vegliante R, Pastushenko I, Blanpain C (2022). Deciphering functional tumor states at single-cell resolution. EMBO J.

[81] Gibbons DL, Lin W, Creighton CJ, Rizvi ZH, Gregory PA, Goodall GJ (2009). Contextual extracellular cues promote tumor cell EMT and metastasis by regulating miR-200 family expression. Genes Dev.

[82] Chen L, Gibbons DL, Goswami S, Cortez MA, Ahn Y-H, Byers LA (2014). Metastasis is regulated via microRNA-200/ZEB1 axis control of tumour cell PD-L1 expression and intratumoral immunosuppression. Nat Commun.

[83] Ye X, Tam WL, Shibue T, Kaygusuz Y, Reinhardt F, Ng Eaton E (2015). Distinct EMT programs control normal mammary stem cells and tumour-initiating cells. Nature.

[84] Dongre A, Rashidian M, Reinhardt F, Bagnato A, Keckesova Z, Ploegh HL (2017). Epithelial-to-Mesenchymal Transition Contributes to Immunosuppression in Breast Carcinomas. Cancer Res.

[85] Dongre A, Rashidian M, Eaton EN, Reinhardt F, Thiru P, Zagorulya M (2021). Direct and Indirect Regulators of Epithelial-Mesenchymal Transition-Mediated Immunosuppression in Breast Carcinomas. Cancer Discov.

[86] Yang L, Zhou Y, Li Y, Zhou J, Wu Y, Cui Y (2015). Mutations of p53 and KRAS activate NF-κB to promote chemoresistance and tumorigenesis via dysregulation of cell cycle and suppression of apoptosis in lung cancer cells. Cancer Lett.

[87] Jinesh GG, Brohl AS (2022). Classical epithelial-mesenchymal transition (EMT) and alternative cell death process-driven blebbishield metastatic-witch (BMW) pathways to cancer metastasis. Signal Transduct Target Ther.

[88] Trimboli AJ, Fukino K, de Bruin A, Wei G, Shen L, Tanner SM (2008). Direct Evidence for Epithelial-Mesenchymal Transitions in Breast Cancer. Cancer Res.

[89] Zhao Z, Zhu X, Cui K, Mancuso J, Federley R, Fischer K (2016). In Vivo Visualization and Characterization of Epithelial–Mesenchymal Transition in Breast Tumors. Cancer Res.

[90] Fischer KR, Durrans A, Lee S, Sheng J, Li F, Wong STC (2015). Epithelial-to-mesenchymal transition is not required for lung metastasis but contributes to chemoresistance. Nature.

[91] Latil M, Nassar D, Beck B, Boumahdi S, Wang L, Brisebarre A (2017). Cell-Type-Specific Chromatin States Differentially Prime Squamous Cell Carcinoma Tumor-Initiating Cells for Epithelial to Mesenchymal Transition. Cell Stem Cell.

[92] Beerling E, Seinstra D, de Wit E, Kester L, van der Velden D, Maynard C (2016). Plasticity between Epithelial and Mesenchymal States Unlinks EMT from Metastasis-Enhancing Stem Cell Capacity. Cell Rep.

[93] Chen Y, LeBleu VS, Carstens JL, Sugimoto H, Zheng X, Malasi S, et al. Dual reporter genetic mouse models of pancreatic cancer identify an epithelial-to-mesenchymal transition-independent metastasis program. EMBO Mol Med. 2018;10:e9085. 10.15252/emmm.201809085 PMC6180301 30120146

[94] Brown MS, Muller KE, Pattabiraman DR (2022). Quantifying the Epithelial-to-Mesenchymal Transition (EMT) from Bench to Bedside. Cancers.

[95] Mak MP, Tong P, Diao L, Cardnell RJ, Gibbons DL, William WN (2016). A Patient-Derived, Pan-Cancer EMT Signature Identifies Global Molecular Alterations and Immune Target Enrichment Following Epithelial-to-Mesenchymal Transition. Clin Cancer Res.

[96] Lou Y, Diao L, Cuentas ERP, Denning WL, Chen L, Fan YH (2016). Epithelial-Mesenchymal Transition Is Associated with a Distinct Tumor Microenvironment Including Elevation of Inflammatory Signals and Multiple Immune Checkpoints in Lung Adenocarcinoma. Clin Cancer Res J Am Assoc Cancer Res.

[97] Chae YK, Chang S, Ko T, Anker J, Agte S, Iams W (2018). Epithelial-mesenchymal transition (EMT) signature is inversely associated with T-cell infiltration in non-small cell lung cancer (NSCLC). Sci Rep.

[98] Wang G, Xu D, Zhang Z, Li X, Shi J, Sun J (2021). The pan-cancer landscape of crosstalk between epithelial-mesenchymal transition and immune evasion relevant to prognosis and immunotherapy response. NPJ Precis Oncol.

[99] Hugo W, Zaretsky JM, Sun L, Song C, Moreno BH, Hu-Lieskovan S (2016). Genomic and Transcriptomic Features of Response to Anti-PD-1 Therapy in Metastatic Melanoma. Cell.

[100] Mariathasan S, Turley SJ, Nickles D, Castiglioni A, Yuen K, Wang Y (2018). TGFβ attenuates tumour response to PD-L1 blockade by contributing to exclusion of T cells. Nature.

[101] Wang L, Saci A, Szabo PM, Chasalow SD, Castillo-Martin M, Domingo-Domenech J (2018). EMT- and stroma-related gene expression and resistance to PD-1 blockade in urothelial cancer. Nat Commun.

[102] Tan TZ, Miow QH, Miki Y, Noda T, Mori S, Huang RY, et al. Epithelial-mesenchymal transition spectrum quantification and its efficacy in deciphering survival and drug responses of cancer patients. EMBO Mol Med. 2014;6:1279–93. 10.15252/emmm.201404208 PMC4287932 25214461

[103] Yoshihara K, Shahmoradgoli M, Martínez E, Vegesna R, Kim H, Torres-Garcia W (2013). Inferring tumour purity and stromal and immune cell admixture from expression data. Nat Commun.

[104] Tyler M, Tirosh I (2021). Decoupling epithelial-mesenchymal transitions from stromal profiles by integrative expression analysis. Nat Commun.

[105] Hara T, Chanoch-Myers R, Mathewson ND, Myskiw C, Atta L, Bussema L (2021). Interactions between cancer cells and immune cells drive transitions to mesenchymal-like states in glioblastoma. Cancer Cell.

[106] Azizi E, Carr AJ, Plitas G, Cornish AE, Konopacki C, Prabhakaran S (2018). Single-Cell Map of Diverse Immune Phenotypes in the Breast Tumor Microenvironment. Cell.

[107] Wu SZ, Al-Eryani G, Roden DL, Junankar S, Harvey K, Andersson A (2021). A single-cell and spatially resolved atlas of human breast cancers. Nat Genet.

[108] Ramachandran P, Dobie R, Wilson-Kanamori JR, Dora EF, Henderson BEP, Luu NT (2019). Resolving the fibrotic niche of human liver cirrhosis at single-cell level. Nature.

[109] Deshmukh AP, Vasaikar SV, Tomczak K, Tripathi S, den Hollander P, Arslan E (2021). Identification of EMT signaling cross-talk and gene regulatory networks by single-cell RNA sequencing. Proc Natl Acad Sci.

[110] McFaline-Figueroa JL, Hill AJ, Qiu X, Jackson D, Shendure J, Trapnell C (2019). A pooled single-cell genetic screen identifies regulatory checkpoints in the continuum of the epithelial-to-mesenchymal transition. Nat Genet.

[111] Sehgal K, Portell AJ, Ivanova EV, Lizotte PH, Mahadevan NR, Greene JR, et al. Dynamic single-cell RNA sequencing identifies immunotherapy persister cells following PD-1 blockade. J Clin Investig. 2021;131:e135038. 10.1172/JCI135038 PMC7810472 33151910

[112] Gangoso E, Southgate B, Bradley L, Rus S, Galvez-Cancino F, McGivern N, et al. Glioblastomas acquire myeloid-affiliated transcriptional programs via epigenetic immunoediting to elicit immune evasion. Cell. 2021;184:2454–70. 10.1016/j.cell.2021.03.023 PMC8099351 33857425

[113] Carstens JL, Yang S, de Sampaio PC, Zheng X, Barua S, McAndrews KM, et al. Stabilized epithelial phenotype of cancer cells in primary tumors leads to increased colonization of liver metastasis in pancreatic cancer. Cell Rep. 2021;35:108990. 10.1016/j.celrep.2021.108990 PMC8078733 33852841

[114] Wu T, Wu X, Wang H-Y, Chen L (2019). Immune contexture defined by single cell technology for prognosis prediction and immunotherapy guidance in cancer. Cancer Commun Lond Engl.

[115] Guruprasad P, Lee YG, Kim KH, Ruella M (2021). The current landscape of single-cell transcriptomics for cancer immunotherapy. J Exp Med.

[116] Ren X, Zhang L, Zhang Y, Li Z, Siemers N, Zhang Z (2021). Insights Gained from Single-Cell Analysis of Immune Cells in the Tumor Microenvironment. Annu Rev Immunol.

[117] Fares CM, Van Allen EM, Drake CG, Allison JP, Hu-Lieskovan S (2019). Mechanisms of Resistance to Immune Checkpoint Blockade: Why Does Checkpoint Inhibitor Immunotherapy Not Work for All Patients?. Am Soc Clin Oncol Educ Book Am Soc Clin Oncol Annu Meet.

[118] Pitt JM, Vétizou M, Daillère R, Roberti MP, Yamazaki T, Routy B (2016). Resistance Mechanisms to Immune-Checkpoint Blockade in Cancer: Tumor-Intrinsic and -Extrinsic Factors. Immunity.

[119] Rizvi H, Sanchez-Vega F, La K, Chatila W, Jonsson P, Halpenny D (2018). Molecular Determinants of Response to Anti–Programmed Cell Death (PD)-1 and Anti–Programmed Death-Ligand 1 (PD-L1) Blockade in Patients With Non–Small-Cell Lung Cancer Profiled With Targeted Next-Generation Sequencing. J Clin Oncol.

[120] Gopalakrishnan V, Helmink BA, Spencer CN, Reuben A, Wargo JA (2018). The Influence of the Gut Microbiome on Cancer, Immunity, and Cancer Immunotherapy. Cancer Cell.

[121] Middha S, Yaeger R, Shia J, Stadler ZK, King S, Guercio S, et al. Majority of B2M-Mutant and -Deficient Colorectal Carcinomas Achieve Clinical Benefit From Immune Checkpoint Inhibitor Therapy and Are Microsatellite Instability-High. JCO Precis Oncol. 2019;3:PO.18.00321. 10.1200/PO.18.00321 PMC6469719 31008436

[122] Gromeier M, Brown MC, Zhang G, Lin X, Chen Y, Wei Z (2021). Very low mutation burden is a feature of inflamed recurrent glioblastomas responsive to cancer immunotherapy. Nat Commun.

[123] McGrail DJ, Pilié PG, Rashid NU, Voorwerk L, Slagter M, Kok M (2021). High tumor mutation burden fails to predict immune checkpoint blockade response across all cancer types. Ann Oncol J Eur Soc Med Oncol.

[124] Platten M, Wick W, Van den Eynde BJ (2012). Tryptophan Catabolism in Cancer: Beyond IDO and Tryptophan Depletion. Cancer Res.

[125] Parajuli G, Tekguc M, Wing JB, Hashimoto A, Okuzaki D, Hirata T (2021). Arid5a Promotes Immune Evasion by Augmenting Tryptophan Metabolism and Chemokine Expression. Cancer Immunol Res.

[126] Lightman SM, Peresie JL, Carlson LM, Holling GA, Honikel MM, Chavel CA (2021). Indoleamine 2,3-dioxygenase 1 is essential for sustaining durable antibody responses. Immunity.

[127] Pérez-Núñez I, Rozalén C, Palomeque JÁ, Sangrador I, Dalmau M, Comerma L (2022). LCOR mediates interferon-independent tumor immunogenicity and responsiveness to immune-checkpoint blockade in triple-negative breast cancer. Nat Cancer.

[128] Hutchison S, Pritchard AL (2018). Identifying neoantigens for use in immunotherapy. Mamm Genome.

[129] Yang P, Meng M, Zhou Q (2021). Oncogenic cancer/testis antigens are a hallmarker of cancer and a sensible target for cancer immunotherapy. Biochim Biophys Acta BBA - Rev Cancer.

[130] Salmaninejad A, Zamani MR, Pourvahedi M, Golchehre Z, Hosseini Bereshneh A, Rezaei N (2016). Cancer/Testis Antigens: Expression, Regulation, Tumor Invasion, and Use in Immunotherapy of Cancers. Immunol Investig.

[131] Akers SN, Odunsi K, Karpf AR (2010). Regulation of cancer germline antigen gene expression: implications for cancer immunotherapy. Future Oncol Lond Engl.

[132] Wang K, Wei G, Liu D (2012). CD19: a biomarker for B cell development, lymphoma diagnosis and therapy. Exp Hematol Oncol.

[133] Barfoed AM, Petersen TR, Kirkin AF, Thor Straten P, Claesson MH, Zeuthen J (2000). Cytotoxic T-lymphocyte clones, established by stimulation with the HLA-A2 binding p5365-73 wild type peptide loaded on dendritic cells In vitro, specifically recognize and lyse HLA-A2 tumour cells overexpressing the p53 protein. Scand J Immunol.

[134] Ellsworth RE, Ellsworth DL, Patney HL, Deyarmin B, Love B, Hooke JA (2008). Amplification of HER2 is a marker for global genomic instability. BMC Cancer.

[135] Bloehdorn J, Braun A, Taylor-Weiner A, Jebaraj BMC, Robrecht S, Krzykalla J (2021). Multi-platform profiling characterizes molecular subgroups and resistance networks in chronic lymphocytic leukemia. Nat Commun.

[136] Shang S, Zhao Y, Qian K, Qin Y, Zhang X, Li T (2022). The role of neoantigens in tumor immunotherapy. Biomed Pharmacother Biomed Pharmacother.

[137] Snyder A, Makarov V, Merghoub T, Yuan J, Zaretsky JM, Desrichard A (2014). Genetic basis for clinical response to CTLA-4 blockade in melanoma. N. Engl J Med.

[138] Rizvi NA, Hellmann MD, Snyder A, Kvistborg P, Makarov V, Havel JJ (2015). Cancer immunology. Mutational landscape determines sensitivity to PD-1 blockade in non-small cell lung cancer. Science.

[139] Josson S, Nomura T, Lin J-T, Huang W-C, Wu D, Zhau HE (2011). β2-microglobulin induces epithelial to mesenchymal transition and confers cancer lethality and bone metastasis in human cancer cells. Cancer Res.

[140] Tu J, Xu H, Ma L, Li C, Qin W, Chen X (2022). Nintedanib enhances the efficacy of PD-L1 blockade by upregulating MHC-I and PD-L1 expression in tumor cells. Theranostics.

[141] Kim S, Koh J, Kim M-Y, Kwon D, Go H, Kim YA (2016). PD-L1 expression is associated with epithelial-to-mesenchymal transition in adenocarcinoma of the lung. Hum Pathol.

[142] Yao J, Caballero OL, Huang Y, Lin C, Rimoldi D, Behren A (2016). Altered Expression and Splicing of ESRP1 in Malignant Melanoma Correlates with Epithelial–Mesenchymal Status and Tumor-Associated Immune Cytolytic Activity. Cancer Immunol Res.

[143] Funaki S, Shintani Y, Fukui E, Yamamoto Y, Kanzaki R, Ose N (2019). The prognostic impact of programmed cell death 1 and its ligand and the correlation with epithelial-mesenchymal transition in thymic carcinoma. Cancer Med.

[144] Hirai M, Kitahara H, Kobayashi Y, Kato K, Bou-Gharios G, Nakamura H (2017). Regulation of PD-L1 expression in a high-grade invasive human oral squamous cell carcinoma microenvironment. Int J Oncol.

[145] Yang J, Tian B, Sun H, Garofalo RP, Brasier AR (2017). Epigenetic silencing of IRF1 dysregulates type III interferon responses to respiratory virus infection in epithelial to mesenchymal transition. Nat Microbiol.

[146] Lee M, Kim DW, Khalmuratova R, Shin S-H, Kim Y-M, Han DH (2019). The IFN-γ-p38, ERK kinase axis exacerbates neutrophilic chronic rhinosinusitis by inducing the epithelial-to-mesenchymal transition. Mucosal Immunol.

[147] Lo U-G, Pong R-C, Yang D, Gandee L, Hernandez E, Dang A (2019). IFNγ-Induced IFIT5 Promotes Epithelial-to-Mesenchymal Transition in Prostate Cancer via miRNA Processing. Cancer Res.

[148] Tseng P-C, Chen C-L, Lee K-Y, Feng P-H, Wang Y-C, Satria RD (2022). Epithelial-to-mesenchymal transition hinders interferon-γ-dependent immunosurveillance in lung cancer cells. Cancer Lett.

[149] Peng W, Chen JQ, Liu C, Malu S, Creasy C, Tetzlaff MT (2016). Loss of PTEN Promotes Resistance to T Cell–Mediated Immunotherapy. Cancer Discov.

[150] Lawson KA, Sousa CM, Zhang X, Kim E, Akthar R, Caumanns JJ (2020). Functional genomic landscape of cancer-intrinsic evasion of killing by T cells. Nature.

[151] Li Z-L, Zhang H-L, Huang Y, Huang J-H, Sun P, Zhou N-N (2020). Autophagy deficiency promotes triple-negative breast cancer resistance to T cell-mediated cytotoxicity by blocking tenascin-C degradation. Nat Commun.

[152] Young TM, Reyes C, Pasnikowski E, Castanaro C, Wong C, Decker CE, et al. Autophagy protects tumors from T cell–mediated cytotoxicity via inhibition of TNFα-induced apoptosis. Sci Immunol. 2020;5:eabb9561. 10.1126/sciimmunol.abb9561 33443027

[153] Akalay I, Janji B, Hasmim M, Noman MZ, Thiery JP, Mami-Chouaib F (2013). EMT impairs breast carcinoma cell susceptibility to CTL-mediated lysis through autophagy induction. Autophagy.

[154] Yaguchi T, Goto Y, Kido K, Mochimaru H, Sakurai T, Tsukamoto N (2012). Immune Suppression and Resistance Mediated by Constitutive Activation of Wnt/β-Catenin Signaling in Human Melanoma Cells. J Immunol.

[155] Spranger S, Bao R, Gajewski TF (2015). Melanoma-intrinsic β-catenin signalling prevents anti-tumour immunity. Nature.

[156] Limagne E, Nuttin L, Thibaudin M, Jacquin E, Aucagne R, Bon M (2022). MEK inhibition overcomes chemoimmunotherapy resistance by inducing CXCL10 in cancer cells. Cancer Cell.

[157] Lamouille S, Xu J, Derynck R (2014). Molecular mechanisms of epithelial–mesenchymal transition. Nat Rev Mol Cell Biol.

[158] Zhang X, Cheng Q, Yin H, Yang G (2017). Regulation of autophagy and EMT by the interplay between p53 and RAS during cancer progression (Review). Int J Oncol.

[159] Su J, Morgani SM, David CJ, Wang Q, Er EE, Huang Y-H (2020). TGF-β orchestrates fibrogenic and developmental EMTs via the RAS effector RREB1. Nature.

[160] Wang Q, Hu B, Hu X, Kim H, Squatrito M, Scarpace L (2017). Tumor Evolution of Glioma-Intrinsic Gene Expression Subtypes Associates with Immunological Changes in the Microenvironment. Cancer Cell.

[161] Qian Y, Yao W, Yang T, Yang Y, Liu Y, Shen Q (2017). aPKC-ι/P-Sp1/Snail signaling induces epithelial–mesenchymal transition and immunosuppression in cholangiocarcinoma. Hepatology.

[162] Katsura A, Tamura Y, Hokari S, Harada M, Morikawa M, Sakurai T (2017). ZEB1‐regulated inflammatory phenotype in breast cancer cells. Mol Oncol.

[163] Guo Y, Lu X, Chen Y, Rendon B, Mitchell RA, Cuatrecasas M (2021). Zeb1 induces immune checkpoints to form an immunosuppressive envelope around invading cancer cells. Sci Adv.

[164] Plaschka M, Benboubker V, Grimont M, Berthet J, Tonon L, Lopez J (2022). ZEB1 transcription factor promotes immune escape in melanoma. J Immunother Cancer.

[165] Munn DH, Mellor AL (2016). IDO in the Tumor Microenvironment: Inflammation, Counter-Regulation, and Tolerance. Trends Immunol.

[166] Kolijn K, Verhoef EI, Smid M, Böttcher R, Jenster GW, Debets R (2018). Epithelial-Mesenchymal Transition in Human Prostate Cancer Demonstrates Enhanced Immune Evasion Marked by IDO1 Expression. Cancer Res.

[167] Ye L-Y, Chen W, Bai X-L, Xu X-Y, Zhang Q, Xia X-F (2016). Hypoxia-Induced Epithelial-to-Mesenchymal Transition in Hepatocellular Carcinoma Induces an Immunosuppressive Tumor Microenvironment to Promote Metastasis. Cancer Res.

[168] Hsu DS-S, Wang H-J, Tai S-K, Chou C-H, Hsieh C-H, Chiu P-H (2014). Acetylation of snail modulates the cytokinome of cancer cells to enhance the recruitment of macrophages. Cancer Cell.

[169] Low-Marchelli JM, Ardi VC, Vizcarra EA, van Rooijen N, Quigley JP, Yang J (2013). Twist1 induces CCL2 and recruits macrophages to promote angiogenesis. Cancer Res.

[170] Wei C, Yang C, Wang S, Shi D, Zhang C, Lin X (2019). Crosstalk between cancer cells and tumor associated macrophages is required for mesenchymal circulating tumor cell-mediated colorectal cancer metastasis. Mol Cancer.

[171] Goyette M-A, Elkholi IE, Apcher C, Kuasne H, Rothlin CV, Muller WJ (2021). Targeting Axl favors an antitumorigenic microenvironment that enhances immunotherapy responses by decreasing Hif-1α levels. Proc Natl Acad Sci USA.

[172] Yang C, Dou R, Wei C, Liu K, Shi D, Zhang C (2021). Tumor-derived exosomal microRNA-106b-5p activates EMT-cancer cell and M2-subtype TAM interaction to facilitate CRC metastasis. Mol Ther J Am Soc Gene Ther.

[173] Kuo C-L, Chou H-Y, Chiu Y-C, Cheng AN, Fan C-C, Chang Y-N (2020). Mitochondrial oxidative stress by Lon-PYCR1 maintains an immunosuppressive tumor microenvironment that promotes cancer progression and metastasis. Cancer Lett.

[174] Taki M, Abiko K, Baba T, Hamanishi J, Yamaguchi K, Murakami R (2018). Snail promotes ovarian cancer progression by recruiting myeloid-derived suppressor cells via CXCR2 ligand upregulation. Nat Commun.

[175] Mao X, Xu J, Wang W, Liang C, Hua J, Liu J (2021). Crosstalk between cancer-associated fibroblasts and immune cells in the tumor microenvironment: new findings and future perspectives. Mol Cancer.

[176] Turley SJ, Cremasco V, Astarita JL (2015). Immunological hallmarks of stromal cells in the tumour microenvironment. Nat Rev Immunol.

[177] Fiori ME, Di Franco S, Villanova L, Bianca P, Stassi G, De Maria R (2019). Cancer-associated fibroblasts as abettors of tumor progression at the crossroads of EMT and therapy resistance. Mol Cancer.

[178] Zhang N, Ng AS, Cai S, Li Q, Yang L, Kerr D (2021). Novel therapeutic strategies: targeting epithelial-mesenchymal transition in colorectal cancer. Lancet Oncol.

[179] Brabletz S, Schuhwerk H, Brabletz T, Stemmler MP. Dynamic EMT: a multi‐tool for tumor progression. EMBO J. 2021;40:e108647. 10.15252/embj.2021108647 PMC8441439 34459003

[180] Wu C-Y, Tsai Y-P, Wu M-Z, Teng S-C, Wu K-J (2012). Epigenetic reprogramming and post-transcriptional regulation during the epithelial–mesenchymal transition. Trends Genet.

[181] Tam WL, Weinberg RA (2013). The epigenetics of epithelial-mesenchymal plasticity in cancer. Nat Med.

[182] Wang Y, Shang Y (2013). Epigenetic control of epithelial-to-mesenchymal transition and cancer metastasis. Exp Cell Res.

[183] Wu Y, Fletcher M, Gu Z, Wang Q, Costa B, Bertoni A (2020). Glioblastoma epigenome profiling identifies SOX10 as a master regulator of molecular tumour subtype. Nat Commun.

[184] Zhang Y, Donaher JL, Das S, Li X, Reinhardt F, Krall JA, et al. Genome-wide CRISPR screen identifies PRC2 and KMT2D-COMPASS as regulators of distinct EMT trajectories that contribute differentially to metastasis. Nat Cell Biol. 2022;24:554–64. 10.1038/s41556-022-00877-0 PMC9037576 35411083

[185] Herranz N, Pasini D, Díaz VM, Francí C, Gutierrez A, Dave N (2008). Polycomb complex 2 is required for E-cadherin repression by the Snail1 transcription factor. Mol Cell Biol.

[186] Sarrió D, Rodriguez-Pinilla SM, Hardisson D, Cano A, Moreno-Bueno G, Palacios J (2008). Epithelial-mesenchymal transition in breast cancer relates to the basal-like phenotype. Cancer Res.

[187] Blick T, Hugo H, Widodo E, Waltham M, Pinto C, Mani SA (2010). Epithelial mesenchymal transition traits in human breast cancer cell lines parallel the CD44(hi/)CD24 (lo/-) stem cell phenotype in human breast cancer. J Mammary Gland Biol Neoplasia.

[188] Chang C-J, Yang J-Y, Xia W, Chen C-T, Xie X, Chao C-H (2011). EZH2 promotes expansion of breast tumor initiating cells through activation of RAF1-β-catenin signaling. Cancer Cell.

[189] Collett K, Eide GE, Arnes J, Stefansson IM, Eide J, Braaten A, et al. Expression of enhancer of zeste homologue 2 is significantly associated with increased tumor cell proliferation and is a marker of aggressive breast cancer. Clin Cancer Res. 2006;12:1168–74. 10.1158/1078-0432.CCR-05-1533 16489070

[190] Kleer CG, Cao Q, Varambally S, Shen R, Ota I, Tomlins SA (2003). EZH2 is a marker of aggressive breast cancer and promotes neoplastic transformation of breast epithelial cells. Proc Natl Acad Sci USA.

[191] McHugh JB, Fullen DR, Ma L, Kleer CG, Su LD (2007). Expression of polycomb group protein EZH2 in nevi and melanoma. J Cutan Pathol.

[192] Zingg D, Debbache J, Schaefer SM, Tuncer E, Frommel SC, Cheng P (2015). The epigenetic modifier EZH2 controls melanoma growth and metastasis through silencing of distinct tumour suppressors. Nat Commun.

[193] Zimmerman SM, Nixon SJ, Chen PY, Raj L, Smith SR, Paolini RL, et al. Ezh2Y641F mutations co-operate with Stat3 to regulate MHC class I antigen processing and alter the tumor immune response in melanoma. Oncogene. 2022;41:4983–93. 10.1038/s41388-022-02492-7 PMC9669177 36220978

[194] Bachmann IM, Halvorsen OJ, Collett K, Stefansson IM, Straume O, Haukaas SA (2006). EZH2 expression is associated with high proliferation rate and aggressive tumor subgroups in cutaneous melanoma and cancers of the endometrium, prostate, and breast. J Clin Oncol J Am Soc Clin Oncol.

[195] von Burstin J, Eser S, Paul MC, Seidler B, Brandl M, Messer M (2009). E-cadherin regulates metastasis of pancreatic cancer in vivo and is suppressed by a SNAIL/HDAC1/HDAC2 repressor complex. Gastroenterology.

[196] Peinado H, Ballestar E, Esteller M, Cano A. Snail mediates E-cadherin repression by the recruitment of the Sin3A/histone deacetylase 1 (HDAC1)/HDAC2 complex. Mol Cell Biol. 2004:24:306–19. 10.1128/MCB.24.1.306-319.2004 PMC303344 14673164

[197] Lin Y, Wu Y, Li J, Dong C, Ye X, Chi Y-I (2010). The SNAG domain of Snail1 functions as a molecular hook for recruiting lysine-specific demethylase 1. EMBO J.

[198] Fu J, Qin L, He T, Qin J, Hong J, Wong J (2011). The TWIST/Mi2/NuRD protein complex and its essential role in cancer metastasis. Cell Res.

[199] Licht JD, Bennett RL (2021). Leveraging epigenetics to enhance the efficacy of immunotherapy. Clin Epigenetics.

[200] Sadagopan A, Michelakos T, Boyiadzis G, Ferrone C, Ferrone S (2022). Human Leukocyte Antigen Class I Antigen-Processing Machinery Upregulation by Anticancer Therapies in the Era of Checkpoint Inhibitors: A Review. JAMA Oncol.

[201] Jones PA, Issa J-PJ, Baylin S (2016). Targeting the cancer epigenome for therapy. Nat Rev Genet.

[202] Gregory PA, Bracken CP, Bert AG, Goodall GJ (2008). MicroRNAs as regulators of epithelial-mesenchymal transition. Cell Cycle Georget Tex.

[203] Gregory PA, Bert AG, Paterson EL, Barry SC, Tsykin A, Farshid G (2008). The miR-200 family and miR-205 regulate epithelial to mesenchymal transition by targeting ZEB1 and SIP1. Nat Cell Biol.

[204] Burk U, Schubert J, Wellner U, Schmalhofer O, Vincan E, Spaderna S (2008). A reciprocal repression between ZEB1 and members of the miR-200 family promotes EMT and invasion in cancer cells. EMBO Rep.

[205] Zhang M, Zhao Z, Pritykin Y, Hannum M, Scott AC, Kuo F (2021). Ectopic activation of the miR-200c-EpCAM axis enhances antitumor T cell responses in models of adoptive cell therapy. Sci Transl Med.

[206] Williams MM, Christenson JL, O’Neill KI, Hafeez SA, Ihle CL, Spoelstra NS (2021). MicroRNA-200c restoration reveals a cytokine profile to enhance M1 macrophage polarization in breast cancer. NPJ Breast Cancer.

[207] Ahn Y-H, Gibbons DL, Chakravarti D, Creighton CJ, Rizvi ZH, Adams HP (2012). ZEB1 drives prometastatic actin cytoskeletal remodeling by downregulating miR-34a expression. J Clin Investig.

[208] Kundu ST, Rodriguez BL, Gibson LA, Warner AN, Perez MG, Bajaj R (2022). The microRNA-183/96/182 cluster inhibits lung cancer progression and metastasis by inducing an interleukin-2-mediated antitumor CD8+ cytotoxic T-cell response. Genes Dev.

[209] Li M, Wang Y, Li M, Wu X, Setrerrahmane S, Xu H (2021). Integrins as attractive targets for cancer therapeutics. Acta Pharm Sin B.

[210] Bagati A, Kumar S, Jiang P, Pyrdol J, Zou AE, Godicelj A (2021). Integrin αvβ6–TGFβ–SOX4 Pathway Drives Immune Evasion in Triple-Negative Breast Cancer. Cancer Cell.

[211] Zhou Y, Slone N, Chrisikos TT, Kyrysyuk O, Babcock RL, Medik YB (2020). Vaccine efficacy against primary and metastatic cancer with in vitro-generated CD103+ conventional dendritic cells. J Immunother Cancer.

[212] Gilles C, Newgreen DF, Sato H, Thompson EW. Matrix Metalloproteases and Epithelial-to-Mesenchymal Transition: Implications for Carcinoma Metastasis. In: Madame Curie Bioscience Database [Internet]. Austin (TX): Landes Bioscience; 2000-2013.

[213] Winkler J, Abisoye-Ogunniyan A, Metcalf KJ, Werb Z (2020). Concepts of extracellular matrix remodelling in tumour progression and metastasis. Nat Commun.

[214] Ye Y, Kuang X, Xie Z, Liang L, Zhang Z, Zhang Y (2020). Small-molecule MMP2/MMP9 inhibitor SB-3CT modulates tumor immune surveillance by regulating PD-L1. Genome Med.

[215] Terry S, Buart S, Tan TZ, Gros G, Noman MZ, Lorens JB (2017). Acquisition of tumor cell phenotypic diversity along the EMT spectrum under hypoxic pressure: Consequences on susceptibility to cell-mediated cytotoxicity. Oncoimmunology.

[216] Akalay I, Tan TZ, Kumar P, Janji B, Mami-Chouaib F, Charpy C, et al. Targeting WNT1-inducible signaling pathway protein 2 alters human breast cancer cell susceptibility to specific lysis through regulation of KLF-4 and miR-7 expression. Oncogene. 2015;34:2261–71. 10.1038/onc.2014.151 24931170

[217] Le Floc’h A, Jalil A, Vergnon I, Le Maux Chansac B, Lazar V, Bismuth G (2007). Alpha E beta 7 integrin interaction with E-cadherin promotes antitumor CTL activity by triggering lytic granule polarization and exocytosis. J Exp Med.

[218] French JJ, Cresswell J, Wong WK, Seymour K, Charnley RM, Kirby JA (2002). T cell adhesion and cytolysis of pancreatic cancer cells: a role for E-cadherin in immunotherapy?. Br J Cancer.

[219] Djenidi F, Adam J, Goubar A, Durgeau A, Meurice G, de Montpréville V (2015). CD8+ CD103+ tumor-infiltrating lymphocytes are tumor-specific tissue-resident memory T cells and a prognostic factor for survival in lung cancer patients. J Immunol Balt Md.

[220] Corgnac S, Damei I, Gros G, Caidi A, Terry S, Chouaib S (2022). Cancer stem-like cells evade CD8+ CD103+ tumor-resident memory T (TRM) lymphocytes by initiating an epithelial-to-mesenchymal transition program in a human lung tumor model. J Immunother Cancer.

[221] Quinn E, Hawkins N, Yip YL, Suter C, Ward R (2003). CD103+ intraepithelial lymphocytes-a unique population in microsatellite unstable sporadic colorectal cancer. Eur J Cancer Oxf Engl.

[222] Wang B, Wu S, Zeng H, Liu Z, Dong W, He W (2015). CD103+ Tumor Infiltrating Lymphocytes Predict a Favorable Prognosis in Urothelial Cell Carcinoma of the Bladder. J Urol.

[223] Abd Hamid M, Colin-York H, Khalid-Alham N, Browne M, Cerundolo L, Chen J-L (2020). Self-Maintaining CD103+ Cancer-Specific T Cells Are Highly Energetic with Rapid Cytotoxic and Effector Responses. Cancer Immunol Res.

[224] Schwartzkopff S, Gründemann C, Schweier O, Rosshart S, Karjalainen KE, Becker K-F (2007). Tumor-associated E-cadherin mutations affect binding to the killer cell lectin-like receptor G1 in humans. J Immunol Balt Md 1950.

[225] Chockley PJ, Chen J, Chen G, Beer DG, Standiford TJ, Keshamouni VG (2018). Epithelial-mesenchymal transition leads to NK cell–mediated metastasis-specific immunosurveillance in lung cancer. J Clin Investig.

[226] Brabletz T, Kalluri R, Nieto MA, Weinberg RA (2018). EMT in cancer. Nat Rev Cancer.

[227] Gonzalez DM, Medici D (2014). Signaling mechanisms of the epithelial-mesenchymal transition. Sci Signal.

[228] Davis FM, Stewart TA, Thompson EW, Monteith GR, Targeting EMT (2014). in cancer: opportunities for pharmacological intervention. Trends Pharm Sci.

[229] Rodon J, Carducci MA, Sepulveda-Sánchez JM, Azaro A, Calvo E, Seoane J (2015). First-in-human dose study of the novel transforming growth factor-β receptor I kinase inhibitor LY2157299 monohydrate in patients with advanced cancer and glioma. Clin Cancer Res J Am Assoc Cancer Res.

[230] Tauriello DVF, Palomo-Ponce S, Stork D, Berenguer-Llergo A, Badia-Ramentol J, Iglesias M (2018). TGFβ drives immune evasion in genetically reconstituted colon cancer metastasis. Nature.

[231] Holmgaard RB, Schaer DA, Li Y, Castaneda SP, Murphy MY, Xu X (2018). Targeting the TGFβ pathway with galunisertib, a TGFβRI small molecule inhibitor, promotes anti-tumor immunity leading to durable, complete responses, as monotherapy and in combination with checkpoint blockade. J Immunother Cancer.

[232] Li L, Wei J-R, Dong J, Lin Q-G, Tang H, Jia Y-X (2021). Laminin γ2-mediating T cell exclusion attenuates response to anti-PD-1 therapy. Sci Adv.

[233] Zhao X, Yang X, Wang X, Zhao X, Zhang Y, Liu S (2021). Penetration Cascade of Size Switchable Nanosystem in Desmoplastic Stroma for Improved Pancreatic Cancer Therapy. ACS Nano.

[234] O’Connor-McCourt MD, Tremblay G, Lenferink A, Sulea T, Zwaagstra J, Koropatnick J (2018). Abstract 1759: AVID200, a highly potent TGF-beta trap, exhibits optimal isoform selectivity for enhancing anti-tumor T-cell activity, without promoting metastasis or cardiotoxicity. Cancer Res.

[235] Yi C, Chen L, Lin Z, Liu L, Shao W, Zhang R (2021). Lenvatinib Targets FGF Receptor 4 to Enhance Antitumor Immune Response of Anti-Programmed Cell Death-1 in HCC. Hepatol Balt Md.

[236] Adachi Y, Kamiyama H, Ichikawa K, Fukushima S, Ozawa Y, Yamaguchi S, et al. Inhibition of FGFR reactivates IFNγ signaling in tumor cells to enhance the combined antitumor activity of lenvatinib with anti-PD-1 antibodies. Cancer Res. 2022:82:292–306. 10.1158/0008-5472.CAN-20-2426 PMC9397636 34753772

[237] Sugiyama E, Togashi Y, Takeuchi Y, Shinya S, Tada Y, Kataoka K (2020). Blockade of EGFR improves responsiveness to PD-1 blockade in EGFR-mutated non-small cell lung cancer. Sci Immunol.

[238] Li C-W, Lim S-O, Xia W, Lee H-H, Chan L-C, Kuo C-W (2016). Glycosylation and stabilization of programmed death ligand-1 suppresses T-cell activity. Nat Commun.

[239] Antony J, Huang RY-J (2017). AXL-Driven EMT State as a Targetable Conduit in Cancer. Cancer Res.

[240] Koorstra J-BM, Karikari CA, Feldmann G, Bisht S, Rojas PL, Offerhaus GJA (2009). The Axl receptor tyrosine kinase confers an adverse prognostic influence in pancreatic cancer and represents a new therapeutic target. Cancer Biol Ther.

[241] Goyette M-A, Duhamel S, Aubert L, Pelletier A, Savage P, Thibault M-P (2018). The Receptor Tyrosine Kinase AXL Is Required at Multiple Steps of the Metastatic Cascade during HER2-Positive Breast Cancer Progression. Cell Rep.

[242] Zhang G, Kong X, Wang M, Zhao H, Han S, Hu R (2018). AXL is a marker for epithelial-mesenchymal transition in esophageal squamous cell carcinoma. Oncol Lett.

[243] Gjerdrum C, Tiron C, Høiby T, Stefansson I, Haugen H, Sandal T (2010). Axl is an essential epithelial-to-mesenchymal transition-induced regulator of breast cancer metastasis and patient survival. Proc Natl Acad Sci USA.

[244] Zhu C, Wei Y, Wei X (2019). AXL receptor tyrosine kinase as a promising anti-cancer approach: functions, molecular mechanisms and clinical applications. Mol Cancer.

[245] Son H-Y, Jeong H-K (2021). Immune Evasion Mechanism and AXL. Front Oncol.

[246] Terry S, Abdou A, Engelsen AST, Buart S, Dessen P, Corgnac S (2019). AXL Targeting Overcomes Human Lung Cancer Cell Resistance to NK- and CTL-Mediated Cytotoxicity. Cancer Immunol Res.

[247] Yokoyama Y, Lew ED, Seelige R, Tindall EA, Walsh C, Fagan PC (2019). Immuno-oncological Efficacy of RXDX-106, a Novel TAM (TYRO3, AXL, MER) Family Small-Molecule Kinase Inhibitor. Cancer Res.

[248] Holtzhausen A, Harris W, Ubil E, Hunter DM, Zhao J, Zhang Y (2019). TAM Family Receptor Kinase Inhibition Reverses MDSC-Mediated Suppression and Augments Anti-PD-1 Therapy in Melanoma. Cancer Immunol Res.

[249] Kasikara C, Davra V, Calianese D, Geng K, Spires TE, Quigley M (2019). Pan-TAM Tyrosine Kinase Inhibitor BMS-777607 Enhances Anti-PD-1 mAb Efficacy in a Murine Model of Triple-Negative Breast Cancer. Cancer Res.

[250] Synn C-B, Kim SE, Lee HK, Kim M-H, Kim JH, Lee JM (2022). SKI-G-801, an AXL kinase inhibitor, blocks metastasis through inducing anti-tumor immune responses and potentiates anti-PD-1 therapy in mouse cancer models. Clin Transl Immunol.

[251] Lee W, Kim DK, Synn C-B, Lee HK, Park S, Jung D-S (2022). Incorporation of SKI-G-801, a Novel AXL Inhibitor, With Anti-PD-1 Plus Chemotherapy Improves Anti-Tumor Activity and Survival by Enhancing T Cell Immunity. Front Oncol.

[252] Boshuizen J, Pencheva N, Krijgsman O, Altimari DD, Castro PG, de Bruijn B (2021). Cooperative Targeting of Immunotherapy-Resistant Melanoma and Lung Cancer by an AXL-Targeting Antibody-Drug Conjugate and Immune Checkpoint Blockade. Cancer Res.

[253] Li L, Han R, Xiao H, Lin C, Wang Y, Liu H (2014). Metformin sensitizes EGFR-TKI-resistant human lung cancer cells in vitro and in vivo through inhibition of IL-6 signaling and EMT reversal. Clin Cancer Res J Am Assoc Cancer Res.

[254] You A, Cao M, Guo Z, Zuo B, Gao J, Zhou H (2016). Metformin sensitizes sorafenib to inhibit postoperative recurrence and metastasis of hepatocellular carcinoma in orthotopic mouse models. J Hematol Oncol J Hematol Oncol.

[255] Deng T, Shen P, Li A, Zhang Z, Yang H, Deng X (2021). CCDC65 as a new potential tumor suppressor induced by metformin inhibits activation of AKT1 via ubiquitination of ENO1 in gastric cancer. Theranostics.

[256] Chou C-C, Lee K-H, Lai I-L, Wang D, Mo X, Kulp SK (2014). AMPK reverses the mesenchymal phenotype of cancer cells by targeting the Akt-MDM2-Foxo3a signaling axis. Cancer Res.

[257] Cha J-H, Yang W-H, Xia W, Wei Y, Chan L-C, Lim S-O (2018). Metformin Promotes Antitumor Immunity via Endoplasmic-Reticulum-Associated Degradation of PD-L1. Mol Cell.

[258] Wen M, Cao Y, Wu B, Xiao T, Cao R, Wang Q (2021). PD-L1 degradation is regulated by electrostatic membrane association of its cytoplasmic domain. Nat Commun.

[259] Scharping NE, Menk AV, Whetstone RD, Zeng X, Delgoffe GM (2017). Efficacy of PD-1 Blockade Is Potentiated by Metformin-Induced Reduction of Tumor Hypoxia. Cancer Immunol Res.

[260] Nishida M, Yamashita N, Ogawa T, Koseki K, Warabi E, Ohue T (2021). Mitochondrial reactive oxygen species trigger metformin-dependent antitumor immunity via activation of Nrf2/mTORC1/p62 axis in tumor-infiltrating CD8T lymphocytes. J Immunother Cancer.

[261] Afzal MZ, Mercado RR, Shirai K (2018). Efficacy of metformin in combination with immune checkpoint inhibitors (anti-PD-1/anti-CTLA-4) in metastatic malignant melanoma. J Immunother Cancer.

[262] Chung SS, Giehl N, Wu Y, Vadgama JV. STAT3 activation in HER2-overexpressing breast cancer promotes epithelial-mesenchymal transition and cancer stem cell traits. Int J Oncol. 2014;44:403–11. 10.3892/ijo.2013.2195 PMC3898805 24297508

[263] Xiong H, Hong J, Du W, Lin Y, Ren L, Wang Y (2012). Roles of STAT3 and ZEB1 proteins in E-cadherin down-regulation and human colorectal cancer epithelial-mesenchymal transition. J Biol Chem.

[264] Yue P, Zhang X, Paladino D, Sengupta B, Ahmad S, Holloway RW (2012). Hyperactive EGF receptor, Jaks and Stat3 signaling promote enhanced colony-forming ability, motility and migration of cisplatin-resistant ovarian cancer cells. Oncogene.

[265] Colomiere M, Ward AC, Riley C, Trenerry MK, Cameron-Smith D, Findlay J (2009). Cross talk of signals between EGFR and IL-6R through JAK2/STAT3 mediate epithelial-mesenchymal transition in ovarian carcinomas. Br J Cancer.

[266] Tam WL, Lu H, Buikhuisen J, Soh BS, Lim E, Reinhardt F (2013). Protein kinase C α is a central signaling node and therapeutic target for breast cancer stem cells. Cancer Cell.

[267] Kawazoe A, Kuboki Y, Shinozaki E, Hara H, Nishina T, Komatsu Y (2020). Multicenter Phase I/II Trial of Napabucasin and Pembrolizumab in Patients with Metastatic Colorectal Cancer (EPOC1503/SCOOP Trial). Clin Cancer Res J Am Assoc Cancer Res.

[268] Rokavec M, Öner MG, Li H, Jackstadt R, Jiang L, Lodygin D (2014). IL-6R/STAT3/miR-34a feedback loop promotes EMT-mediated colorectal cancer invasion and metastasis. J Clin Investig.

[269] Hahn S, Jackstadt R, Siemens H, Hünten S, Hermeking H (2013). SNAIL and miR-34a feed-forward regulation of ZNF281/ZBP99 promotes epithelial-mesenchymal transition. EMBO J.

[270] Cortez MA, Ivan C, Valdecanas D, Wang X, Peltier HJ, Ye Y (2016). PDL1 Regulation by p53 via miR-34. J Natl Cancer Inst.

[271] Shah P, Gau Y, Sabnis G (2014). Histone deacetylase inhibitor entinostat reverses epithelial to mesenchymal transition of breast cancer cells by reversing the repression of E-cadherin. Breast Cancer Res Treat.

[272] Song X, Wang J, Zheng T, Song R, Liang Y, Bhatta N (2013). LBH589 Inhibits proliferation and metastasis of hepatocellular carcinoma via inhibition of gankyrin/STAT3/Akt pathway. Mol Cancer.

[273] Wu R, Wang C, Li Z, Xiao J, Li C, Wang X (2020). SOX2 promotes resistance of melanoma with PD-L1 high expression to T-cell-mediated cytotoxicity that can be reversed by SAHA. J Immunother Cancer.

[274] Zheng H, Zhao W, Yan C, Watson CC, Massengill M, Xie M (2016). HDAC Inhibitors Enhance T-Cell Chemokine Expression and Augment Response to PD-1 Immunotherapy in Lung Adenocarcinoma. Clin Cancer Res J Am Assoc Cancer Res.

[275] Li M, Wang Y, Li M, Wu X, Setrerrahmane S, Xu H (2021). Integrins as attractive targets for cancer therapeutics. Acta Pharm Sin B.

[276] Ye Y, Kuang X, Xie Z, Liang L, Zhang Z, Zhang Y (2020). Small-molecule MMP2/MMP9 inhibitor SB-3CT modulates tumor immune surveillance by regulating PD-L1. Genome Med.

[277] Zhao F, Evans K, Xiao C, DeVito N, Theivanthiran B, Holtzhausen A (2018). Stromal Fibroblasts Mediate Anti-PD-1 Resistance via MMP-9 and Dictate TGFβ Inhibitor Sequencing in Melanoma. Cancer Immunol Res.

[278] Strauss J, Gatti-Mays ME, Cho BC, Hill A, Salas S, McClay E (2020). Bintrafusp alfa, a bifunctional fusion protein targeting TGF-β and PD-L1, in patients with human papillomavirus-associated malignancies. J Immunother Cancer.

[279] Gachpazan M, Kashani H, Hassanian SM, Khazaei M, Khorrami S, Ferns GA (2019). Therapeutic Potential of Targeting Transforming Growth Factor-beta in Colorectal Cancer: Rational and Progress. Curr Pharm Des.

[280] Hsu C, Chang Y-F, Yen C-J, Lu L-C, Zhu X, Xu Y (2021). Safety and efficacy of combination of GT90001, an anti-activin receptor-like kinase-1 (ALK-1) antibody, and nivolumab in patients with metastatic hepatocellular carcinoma (HCC). J Clin Oncol.

[281] Percent IJ, Reynolds CH, Konduri K, Whitehurst MT, Nidhiry EA, Yanagihara RH (2020). Phase III trial of sitravatinib plus nivolumab vs. docetaxel for treatment of NSCLC after platinum-based chemotherapy and immunotherapy (SAPPHIRE). J Clin Oncol.

[282] Zhao Y, Zhang Y-N, Wang K-T, Chen L (2020). Lenvatinib for hepatocellular carcinoma: From preclinical mechanisms to anti-cancer therapy. Biochim Biophys Acta Rev Cancer.

[283] Kubli SP, Berger T, Araujo DV, Siu LL, Mak TW. Beyond immune checkpoint blockade: emerging immunological strategies. Nat Rev Drug Discov. 2021:20:899–919. 10.1038/s41573-021-00155-y 33686237

[284] Patel SA, Minn AJ (2018). Combination Cancer Therapy with Immune Checkpoint Blockade: Mechanisms and Strategies. Immunity.

[285] Weng Y-S, Tseng H-Y, Chen Y-A, Shen P-C, Al Haq AT, Chen L-M (2019). MCT-1/miR-34a/IL-6/IL-6R signaling axis promotes EMT progression, cancer stemness and M2 macrophage polarization in triple-negative breast cancer. Mol Cancer.

[286] Lin X, Wang S, Sun M, Zhang C, Wei C, Yang C (2019). miR-195-5p/NOTCH2-mediated EMT modulates IL-4 secretion in colorectal cancer to affect M2-like TAM polarization. J Hematol Oncol J Hematol Oncol.

[287] Sami E, Paul BT, Koziol JA, ElShamy WM (2020). The Immunosuppressive Microenvironment in BRCA1-IRIS-Overexpressing TNBC Tumors Is Induced by Bidirectional Interaction with Tumor-Associated Macrophages. Cancer Res.

[288] Wang T, Jing B, Xu D, Liao Y, Song H, Sun B (2020). PTGES/PGE2 signaling links immunosuppression and lung metastasis in Gprc5a-knockout mouse model. Oncogene.

[289] Yoon J-H, Jung SM, Park SH, Kato M, Yamashita T, Lee I-K (2013). Activin receptor-like kinase5 inhibition suppresses mouse melanoma by ubiquitin degradation of Smad4, thereby derepressing eomesodermin in cytotoxic T lymphocytes. EMBO Mol Med.

[290] Zizzari IG, Napoletano C, Botticelli A, Caponnetto S, Calabrò F, Gelibter A (2018). TK Inhibitor Pazopanib Primes DCs by Downregulation of the β-Catenin Pathway. Cancer Immunol Res.

[291] Bedke J, Albiges L, Capitanio U, Giles RH, Hora M, Lam TB (2021). Updated European Association of Urology Guidelines on Renal Cell Carcinoma: Nivolumab plus Cabozantinib Joins Immune Checkpoint Inhibition Combination Therapies for Treatment-naïve Metastatic Clear-Cell Renal Cell Carcinoma. Eur Urol.

[292] Lu X, Horner JW, Paul E, Shang X, Troncoso P, Deng P (2017). Effective combinatorial immunotherapy for castration-resistant prostate cancer. Nature.

[293] Tsukita Y, Fujino N, Miyauchi E, Saito R, Fujishima F, Itakura K (2019). Axl kinase drives immune checkpoint and chemokine signalling pathways in lung adenocarcinomas. Mol Cancer.

[294] Sadahiro H, Kang K-D, Gibson JT, Minata M, Yu H, Shi J (2018). Activation of the Receptor Tyrosine Kinase AXL Regulates the Immune Microenvironment in Glioblastoma. Cancer Res.

[295] Seifert AM, Zeng S, Zhang JQ, Kim TS, Cohen NA, Beckman MJ (2017). PD-1/PD-L1 Blockade Enhances T-cell Activity and Antitumor Efficacy of Imatinib in Gastrointestinal Stromal Tumors. Clin Cancer Res J Am Assoc Cancer Res.

[296] Liu P, Zhao L, Pol J, Levesque S, Petrazzuolo A, Pfirschke C (2019). Crizotinib-induced immunogenic cell death in non-small cell lung cancer. Nat Commun.

[297] Oh CY, Klatt MG, Bourne C, Dao T, Dacek MM, Brea EJ (2019). ALK and RET Inhibitors Promote HLA Class I Antigen Presentation and Unmask New Antigens within the Tumor Immunopeptidome. Cancer Immunol Res.

[298] Wei Z, Zhang X, Yong T, Bie N, Zhan G, Li X (2021). Boosting anti-PD-1 therapy with metformin-loaded macrophage-derived microparticles. Nat Commun.

[299] Lin J, Sampath D, Nannini MA, Lee BB, Degtyarev M, Oeh J (2013). Targeting activated Akt with GDC-0068, a novel selective Akt inhibitor that is efficacious in multiple tumor models. Clin Cancer Res J Am Assoc Cancer Res.

[300] Oki Y, Buglio D, Zhang J, Ying Y, Zhou S, Sureda A (2014). Immune regulatory effects of panobinostat in patients with Hodgkin lymphoma through modulation of serum cytokine levels and T-cell PD1 expression. Blood Cancer J.

